# Comparison of the Melbourne rapid fields-web device-independent online perimeter and the octopus 600 perimeter in glaucoma patients

**DOI:** 10.3389/fopht.2026.1961375

**Published:** 2026-10-12

**Authors:** George Y. X. Kong, Algis Vingrys, Manisha Ambawat, Md Mahboob, Shakira Bano, Syril Dorairaj, Shibal Bhartiya

**Affiliations:** 1 The University of Melbourne, Parkville, VIC, Australia; 2 Centre for Eye Research Australia, East Melbourne, VIC, Australia; 3 Royal Victorian Eye and Ear Hospital, East Melbourne, VIC, Australia; 4 Marengo Asia Hospitals, Gurugram, Haryana, India; 5 Department of Ophthalmology, Mayo Clinic, Jacksonville, FL, United States

**Keywords:** glaucoma, Melbourne rapid fields, octopus perimeter, online perimetry, visual field

## Abstract

**Introduction:**

The purpose of this study is to evaluate the agreement, repeatability, and testing efficiency of Melbourne Rapid Fields-Web (MRF-Web), a browser-based, device-independent perimeter, compared with the Octopus 600 perimeter in glaucoma patients.

**Methods:**

This retrospective, cross-sectional, single-center observational study included 113 patients undergoing routine visual field assessment for glaucoma or glaucoma suspicion at Marengo Asia Hospitals, Gurgaon, India. MRF-Web testing was performed on a calibrated 14-inch laptop with Hindi voice guidance. Agreement between MRF-Web and Octopus 600 for Mean Deviation (MD) and pattern-based global indices was assessed using intraclass correlation coefficients (ICCs) and Bland–Altman analysis. Test-retest repeatability was evaluated in 51 patients who underwent repeat MRF-Web testing.

**Results:**

MRF-Web showed strong group-level concordance with Octopus 600 for MD, with an ICC of 0.88 (95% CI, 0.81–0.92), Bland–Altman bias of -0.78 dB, and 95% limits of agreement from -4.72 to 3.17 dB. Concordance for the pattern-based indices was moderate, with an ICC of 0.72 (95% CI, 0.58–0.81), bias of -0.59 dB, and 95% limits of agreement from -4.41 to 3.24 dB. MRF-Web demonstrated good test-retest concordance, with an ICC of 0.86 (95% CI, 0.69–0.94). Mean per-eye test duration was significantly shorter with MRF-Web than with Octopus 600 (232.9 ± 49.8 versus 342.4 ± 94.4 seconds).

**Conclusions:**

MRF-Web demonstrated strong group-level concordance with Octopus 600 MD measurements and shorter per-eye testing times in this predominantly mild-disease cohort. The limits of agreement indicate that measurements from the two platforms should not be regarded as directly interchangeable, and further study is required in moderate and advanced glaucoma.

## Introduction

Glaucoma is a leading cause of irreversible blindness worldwide (1, 2). The disease is characterized by progressive retinal ganglion cell loss (3) and functional damage as captured by characteristic visual field defects. Accordingly, perimetry is central to diagnosis, staging, and longitudinal monitoring (4, 5). Because glaucomatous field loss may remain asymptomatic, improving access to timely functional testing remains a public-health and service-delivery priority (4, 6).

Longitudinal care requires repeated testing to detect progression and guide treatment escalation (7). However, standard automated perimeters are expensive, non-portable, and require dedicated space and trained staff, factors that can reduce testing frequency and limit uptake, especially in high-volume clinics and resource-constrained regions (6, 8). Technology-enabled strategies, including tablet-, computer-screen-, and headset-based perimetry, have therefore been proposed to expand access without compromising clinical utility (8–10).

Melbourne Rapid Fields (MRF) (www.mrfvision.com) was developed as a portable perimetry platform and has been validated against conventional perimetry using the Humphrey Field Analyzer (HFA), with favorable concordance and repeatability (10–14). MRF incorporates stimulus scaling with eccentricity to reduce variability and maintain usable dynamic range in the periphery or with mild to moderate loss (15) and clinical evidence shows that larger stimuli can reduce variability in damaged fields without sacrificing detection capability (16, 17).

In 2020, MRF was translated from the previous iPad app to a device-independent web-browser application (MRF-Web) to facilitate testing on commonly available computers and to support remote monitoring (10). Telemedicine applications are increasingly relevant for glaucoma, with evidence that more frequent testing can identify meaningful change earlier (18) and that sustained home testing is feasible for many patients (19, 20). MRF-Web has shown promising performance in controlled and home environments (21), and recent studies have reported real-world usability and clinical applicability in India (22), comparative performance in Chinese glaucoma cohorts (11), and additional international validation across testing patterns and settings (10, 23–25).

The Octopus 600 perimeter (Haag-Streit Diagnostics, Köniz, Switzerland) is widely used in clinical practice and provides a relevant comparator for assessing whether a browser-based approach can match conventional clinical output while improving efficiency. The present study therefore evaluates the test times and agreement between MRF-Web and Octopus 600 perimetry in a single-center Indian clinical cohort using a 14-inch laptop with Hindi voice guidance. In this study, we also quantify MRF-Web test-retest repeatability in a subset of participants.

## Materials and methods

### Study design

This retrospective, cross-sectional, single-center observational study was conducted at Marengo Asia Hospitals, Gurgaon, India. De-identified clinical perimetry data collected during routine care were analyzed in accordance with institutional governance and the Declaration of Helsinki.

### Participants

Patients undergoing routine visual field assessment for glaucoma or glaucoma suspicion were eligible for inclusion. All eyes had a visual acuity of 6/12 (20/40) or better. Participants were excluded if they had intraocular surgery 6 months before MRF visual field testing. Lens status was not a criterion for exclusion, provided that any lens opacity did not reduce visual acuity below the 6/12 (20/40) inclusion criterion. Patients with a spherical equivalent (SE) of ≤ −8 D (myopia) or ≥ +6 D (hypermetropia) were excluded from recruitment.

Where both eyes were tested, a single study eye was selected for descriptive summaries to avoid within-participant correlation (right eye by default, unless it does not meet inclusion criteria); for agreement analyses reported in the main outcomes, eyes meeting predefined quality criteria were included as per the study dataset (n = 113). A subset of 51 patients returned within 3 months for repeat MRF testing to assess test-retest reliability. For 48 of the 51 participants, repeat MRF-Web testing was performed during the same clinical session following an approximately 10-minute rest period. Three participants were unable to complete the repeat test during the same session: two returned approximately 1 month later and one returned 2 months later.

### Perimetry procedures

MRF-Web perimetry has been described previously (10, 11, 24). In brief, a 24-2 grid stimulus pattern is used for testing and threshold levels are estimated using a rapid neighborhood-based Zippy Estimation by Sequential Testing (ZEST) approach with seven discrete threshold steps over a 0–30 dB range. Stimulus size is scaled with eccentricity to reduce variability and return approximately constant thresholds across the field (15), consistent with known spatial summation behavior and clinical evidence supporting the benefits of larger peripheral stimuli (17). Participants respond via keyboard input, and Hindi voice prompts guide test progression.

In this study, all MRF-Web tests were performed on a 14-inch laptop with brightness set to maximum and screen calibration completed using the in-software calibration routine before testing. Hindi voice guidance was enabled for all participants to standardize instruction and reduce language-related barriers, aligning with real-world implementation priorities in diverse populations (22). All testing was conducted in the clinic in a darkened room and supervised by a trained technician. The devices accessed the MRF-Web browser application using the Google Chrome browser. Calibration involves the display of an inverted U-shaped virtual ruler, which participants adjust to match the width of a standard credit card (53.98 mm), allowing for the calculation of screen resolution and size. For the 14-inch screen, the viewing distance was 33 cm. The brightness of the screen was set to maximum (350 cd m^-2^). The fellow eye was occluded and the participants’ habitual near correction was used where required. Testing was conducted in a darkened room to minimize glare. All calibration and brightness adjustments were performed by the clinical assistant before testing patients. The clinical assistant also detailed the test procedure to the patient and voice prompts were given in Hindi by the device during the test to guide the user through the test. Additionally, the webcam of the device was used for facial tracking to continuously monitor the viewing distance throughout the test by comparing the present size of the face to the size set at the desired viewing distance at the start of the test. Participants were alerted to changes of over 5% in viewing distance with a moving animation at the fixation target, and at a 10% change in viewing distance, a computer-generated voice command prompted them to readjust the distance from the screen. Fixation monitoring was performed using a standard blind-spot monitoring technique. The camera feed was also analyzed in real time to assess eye gaze stability, and gaze was plotted on the perimeter report. Testing was performed with natural pupils in dim room lighting. The order of Octopus and MRF testing followed routine clinic workflows.

For MRF-Web, Mean Deviation (MD) was calculated from the average point-wise deviations relative to the age-adjusted expected threshold derived from analysis of 17,390 thresholds from normal and glaucoma participants, with adjustment for test-grid location and stimulus-size effects. Pattern Deviation (PD) was calculated using standard formulas as the average residual deviation after accounting for the patient’s MD. Unlike HFA, there is no proprietary adjustment or weighting of values based on test-point eccentricity; therefore, MRF does not use the nomenclature of Pattern Standard Deviation (PSD). Octopus 600 testing was performed using the 24–2 program with the Dynamic thresholding strategy. Results were analyzed using the EyeSuite (version i9.16.2.0) HFA-style Single Field Analysis output. MD and PSD values were taken directly from the EyeSuite-generated HFA-style report. EyeSuite performs the conversion from native Octopus output to HFA-style indices, including the corresponding MD sign convention, using proprietary manufacturer software, and the exact transformation formulas are not publicly available. No additional sign conversion or recalculation was performed by the investigators.

### Outcomes and statistical analysis

All analyses were done using SPSS (version 29.0.1.0; IBM Corp., Armonk, NY, USA) or GraphPad Prism v10.1.1 for Mac (GraphPad Software, Boston, MA, USA). Intraclass correlation coefficients (ICCs) were used to report concordance between the two devices for Mean Deviation (MD) and pattern-based global indices (Pattern Deviation [PD] on MRF and Pattern Standard Deviation [PSD] on Octopus 600). Bland–Altman analysis was undertaken to identify bias and 95% limits of agreement (LoA) when comparing Octopus 600 and MRF outcomes. Paired t-tests or Wilcoxon signed-rank tests were used for within-participant comparisons, as appropriate according to the distribution of the paired differences, with statistical significance set at p < 0.05. A paired t-test was used for normally distributed paired differences, while the Wilcoxon signed-rank test was used when the paired differences were not normally distributed.

## Results

### Participant demographics and baseline characteristics

A total of 113 participants were included in this study. The average age of the patients was 56.5 ± 16.6 years, with 63 male and 50 female participants (**Table 1**). Visual field defect severity in the study eye, based on the Hodapp–Parrish–Anderson classification of the Octopus 600 MD value, is listed in **Table 1**; most cases were mild (n = 103), with five cases in the moderate category and five cases in the severe category.

**Table 1:** Patient demographics of the study cohort.

Characteristic	Value
Participants	113
Sex (male/female)	63/50
Age (years), mean ± SD	56.5 ± 16.6
Visual field severity	
Mild (MD > -6 dB)	103
Moderate (-12 < MD ≤ -6 dB)	5
Severe (MD ≤ -12 dB)	5

SD, standard deviation; MD, Mean Deviation.

### Agreement between MRF-Web and Octopus 600

MRF-Web demonstrated strong group-level concordance with Octopus 600 for MD (**Figure 1**). The ICC(2,1) for MD was 0.88 (95% CI 0.81–0.92). Bland–Altman analysis showed 95% limits of agreement (LoA) of -4.72 to +3.17 dB with a bias of -0.78 dB. The 95% CI for the mean bias was −1.15 to −0.40 dB.

**Figure 1:**
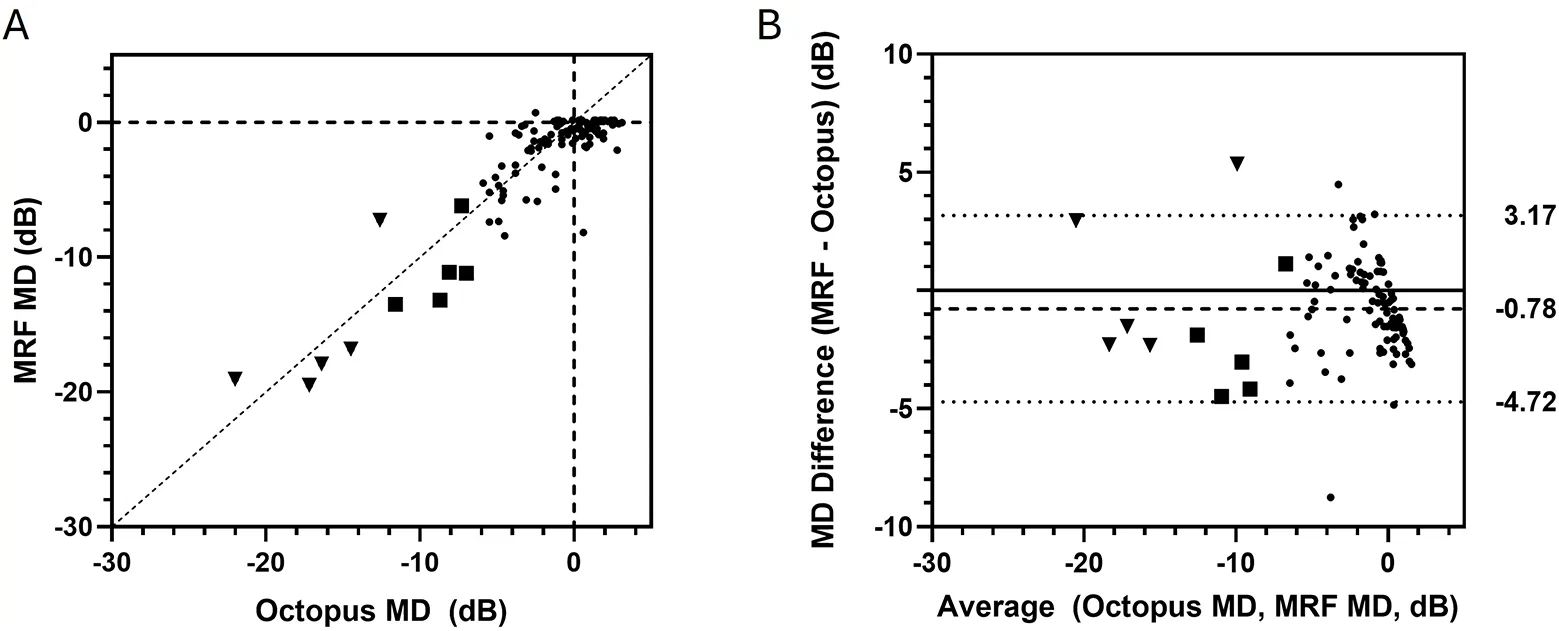
**(A)** Correlation between Mean Deviation (MD) values from Octopus and Melbourne Rapid Fields (MRF) devices; **(B)** Bland–Altman analysis comparing MD values from Octopus and MRF devices (n = 113). Circles indicate cases of mild disease, squares indicate cases of moderate disease, and triangles indicate cases of severe disease.

For the pattern-based global indices, MRF-Web showed moderate concordance with Octopus 600, with an ICC(2,1) of 0.72 (95% CI: 0.58 to 0.81) (**Figure 2**). Bland–Altman analysis showed 95% LoA of -4.41 to +3.24 dB with a bias of -0.59 dB.

**Figure 2:**
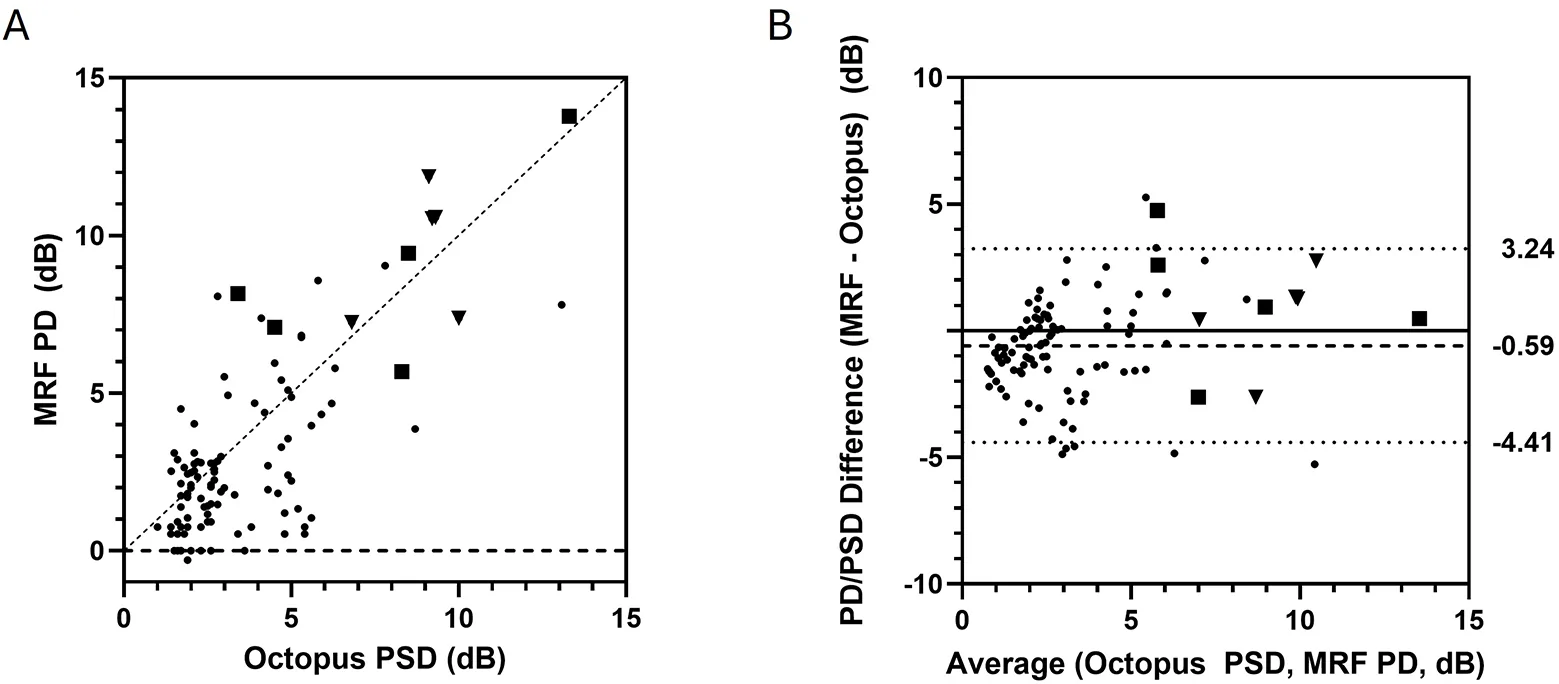
**(A)** Correlation between Pattern Standard Deviation (PSD) values from Octopus and Pattern Deviation (PD) values from Melbourne Rapid Fields (MRF); **(B)** Bland–Altman analysis comparing PSD values from Octopus and PD values from MRF (n = 113). Circles indicate cases of mild disease, squares indicate cases of moderate disease, and triangles indicate cases of severe disease.

### MRF test-retest repeatability

In the subset of patients (n = 51), MRF testing was repeated within 3 months of the first test. The severity distribution of this test-retest group, based on the Hodapp–Parrish–Anderson classification of the Octopus 600 MD value, included 44 cases of mild glaucoma, three cases of moderate glaucoma, and four cases of severe glaucoma. Comparison of MRF MD values from test 1 and test 2 showed good group-level concordance with an ICC(2,1) of 0.86 (95% CI 0.69–0.94) (**Figure 3**). Bland–Altman analysis showed 95% LoA of -3.57 to +5.20 dB with a bias of +0.81 dB. The test-retest difference was significantly associated with the mean MD of the two tests (Pearson r = −0.53, p < 0.001; linear regression slope −0.275, 95% CI −0.401 to −0.148; R² = 0.28), indicating greater positive test-retest differences with increasing visual field loss.

**Figure 3:**
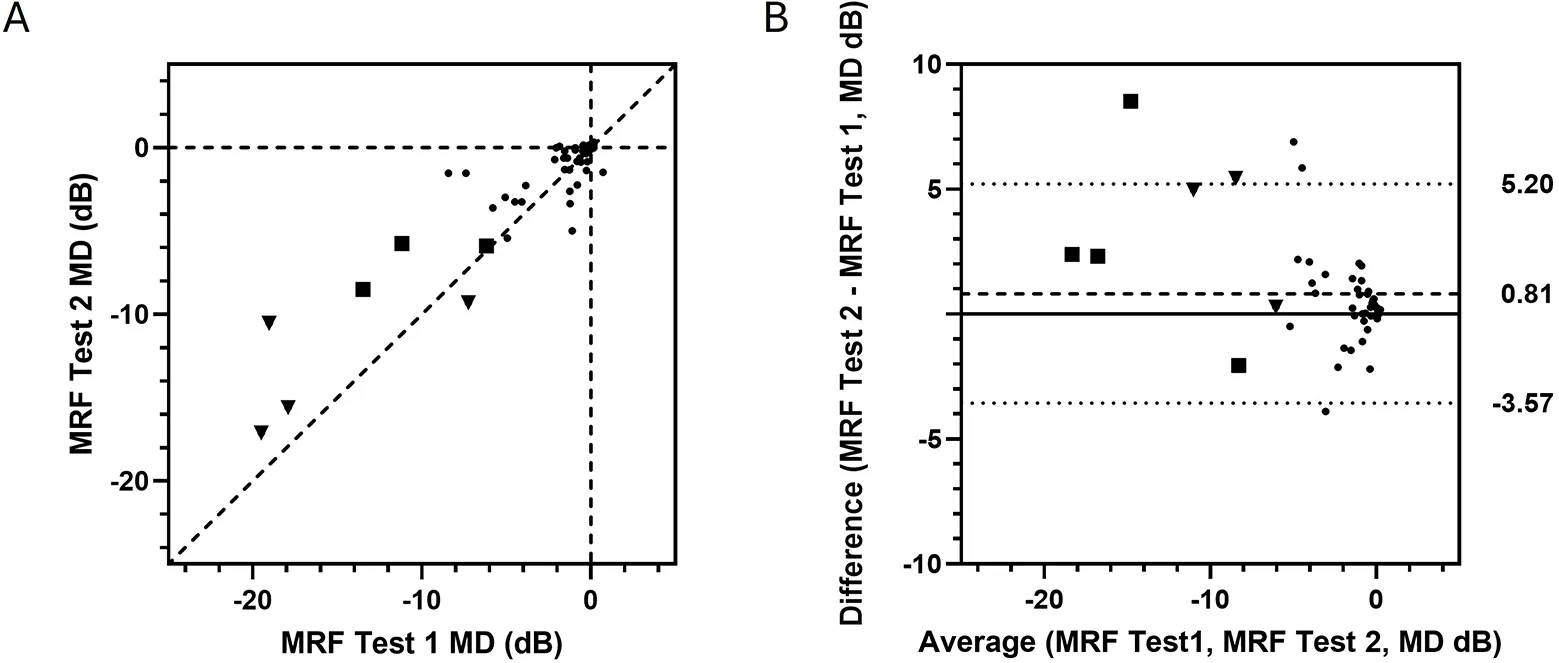
**(A)** Correlation between Mean Deviation (MD) values from Melbourne Rapid Fields (MRF) tests 1 and 2; **(B)** Bland–Altman analysis comparing MD values from MRF tests 1 and 2 (n = 51). Circles indicate cases of mild disease, squares indicate cases of moderate disease, and triangles indicate cases of severe disease.

### Efficiency and clinical throughput

There was a substantial reduction in test time with MRF-Web compared to Octopus 600. The average per-eye testing time for Octopus 600 was 342.4 ± 94.4 seconds (5.7 min), compared with 232.9 ± 49.8 seconds (3.9 min) per eye for MRF. MRF was approximately 1.8 minutes faster per eye than Octopus 600. The false-positive error rate was higher with the MRF test than with Octopus 600 (4.9% vs. 0.78%; p = 0.001). There was no statistically significant difference in false-negative errors between the two devices (**Table 2**). A comparison of fixation loss is not possible because Octopus 600 does not record fixation-loss error measurements.

**Table 2:** Overall baseline global indices and test duration for the study eye of each participant in this study.

Measure	Octopus 600	MRF-Web	P
MD (dB), mean ± SD	-1.75 ± 4.28	-2.51 ± 4.19	< 0.01
PSD/PD (dB), mean ± SD	3.68 ± 2.41	3.05 ± 2.89	0.02
Test duration (s), mean ± SD	342.4 ± 94.4	232.9 ± 49.8	< 0.01
False-positive errors	0.78% (0%, 40%)	4.9% (0%, 83%)	0.001
False-negative errors	4.1% (0%, 30%)	4.5% (0%, 62%)	0.88

SD, standard deviation; MD, Mean Deviation; PSD, Pattern Standard Deviation; PD, Pattern Deviation.

## Discussion

In this single-center clinical cohort, MRF-Web demonstrated strong group-level concordance with Octopus 600 for MD, moderate concordance for the pattern-based global indices, and a shorter mean per-eye test duration. The MD ICC of 0.88 (95% CI 0.81–0.92) reflects strong relative concordance, but the 95% LoA of -4.72 to +3.17 dB and a bias of -0.78 dB indicate a small but statistically significant systematic tendency toward more negative MD values with MRF-Web. Therefore, the two devices should not be considered directly interchangeable for individual measurements, and serial measurements should be interpreted using the same perimetric platform.

For repeat MRF testing, the ICC of 0.86 (95% CI 0.69–0.94) was accompanied by 95% LoA of -3.57 to +5.20 dB. Test-retest differences were significantly associated with mean MD, with eyes showing greater visual field loss tending to have more positive MD values on repeat testing. The improvement observed in several eyes on the second test may represent learning or severity-dependent variability; however, only seven participants in the repeatability subgroup had moderate or severe field loss, precluding reliable severity-stratified estimates of repeatability. Therefore, the present findings support overall repeatability at a group level but do not establish suitability for longitudinal progression monitoring.

The moderate ICC(2,1) of 0.72 (95% CI: 0.58 to 0.81) for the pattern-based indices and the bias of -0.59 dB should be interpreted in the context of device-specific analytical methods. MRF uses a neighborhood-based ZEST thresholding strategy, seven discrete threshold levels over a 0–30 dB range, eccentricity-scaled stimuli, and its own normative framework, whereas the Octopus output is generated using its device-specific acquisition and analytical system. The HFA-style Octopus report format should not be taken to indicate identity of the underlying measurements. Thus, the two pattern-based indices are clinically analogous summary measures rather than mathematically identical quantities. Although MRF-Web and the EyeSuite HFA-style Octopus output both provide global indices describing generalized and localized field loss, these indices are device-specific and should not be considered mathematically equivalent. Accordingly, the ICC and Bland–Altman analyses of pattern-based indices in this study quantify concordance between related device-specific summary measures rather than establish direct numerical interchangeability.

The principal efficiency finding was that MRF was approximately 1.8 minutes faster per tested eye than Octopus 600. Shorter per-eye test duration may reduce patient burden and improve service efficiency in high-volume settings. Because bilateral testing time was not directly evaluated as an outcome in this study, no bilateral time-saving estimate can be inferred from these data.

In the present study, MRF-Web was evaluated under standardized clinic conditions using a single calibrated 14-inch laptop, supervised testing, and Hindi voice guidance. This configuration demonstrates feasibility under these specific testing conditions but does not establish equivalent performance across different screen types, languages, or clinical environments. Previous studies have evaluated MRF on several computer devices and in different populations; however, such evidence was based on comparisons with the Humphrey Field Analyzer and should be considered separately from the findings of the present study (10, 11, 22, 24, 26). Further study will be required to evaluate MRF and Octopus 600 across a broader range of devices and patient cohorts.

This study has several limitations. First, because this was a single-center retrospective analysis, selection and workflow factors may influence generalizability. Second, the cohort was markedly weighted toward mild visual field loss: 103 of 113 participants were classified as mild, with only five moderate and five severe cases. The agreement estimates therefore primarily characterize performance in mild disease and should not be generalized to moderate or advanced glaucoma. The small number of more advanced cases also precluded meaningful severity-stratified statistical analysis. Third, while the dataset includes repeat MRF testing in 51 patients, repeat testing with Octopus 600 was not performed, limiting direct comparison of device-specific repeatability. The relatively wide MRF test-retest LoA also indicate that appreciable within-eye variability may occur, particularly in eyes with moderate or severe disease, and the present study does not provide sufficient evidence of performance for longitudinal progression detection. Finally, testing was performed using a single calibrated 14-inch laptop in one supervised clinical environment; performance across different screens, languages, and testing environments was not assessed in this study and requires further investigation (27).

In conclusion, MRF-Web demonstrated strong group-level concordance with Octopus 600 MD measurements and significantly shorter per-eye testing time in this predominantly mild-disease cohort. The inter-device and test-retest limits of agreement indicate that clinically relevant differences can occur in individual eyes, and measurements from different platforms should not be considered directly interchangeable. Further studies including larger numbers of moderate and advanced glaucoma cases are required to establish performance across the full range of visual field loss and for longitudinal monitoring.

## Data Availability

The original contributions presented in the study are included in the article/**Supplementary Material**. Further inquiries can be directed to the corresponding author.
