# Supplementary material for: Comparison of the Melbourne rapid fields-web device-independent online perimeter and the octopus 600 perimeter in glaucoma patients
Source: Front Ophthalmol (Lausanne). 2026 Oct 12;6:1961375. doi: 10.3389/fopht.2026.1961375 (PMC13697594; doi:10.3389/fopht.2026.1961375)

Example Octopus 600 vs MRF outputs

Severe Glaucoma Case 1

Octopus 600

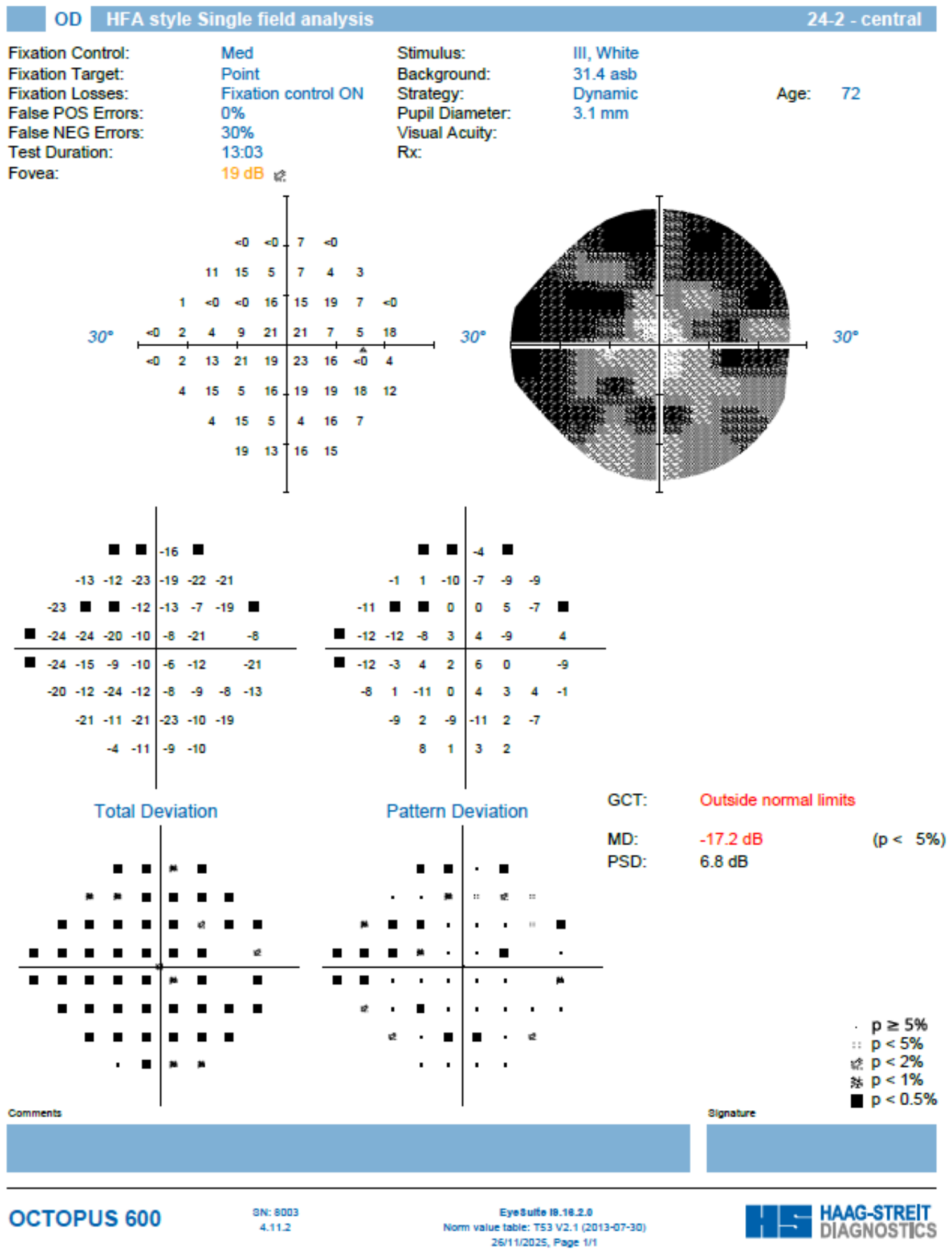

MRF Test 1

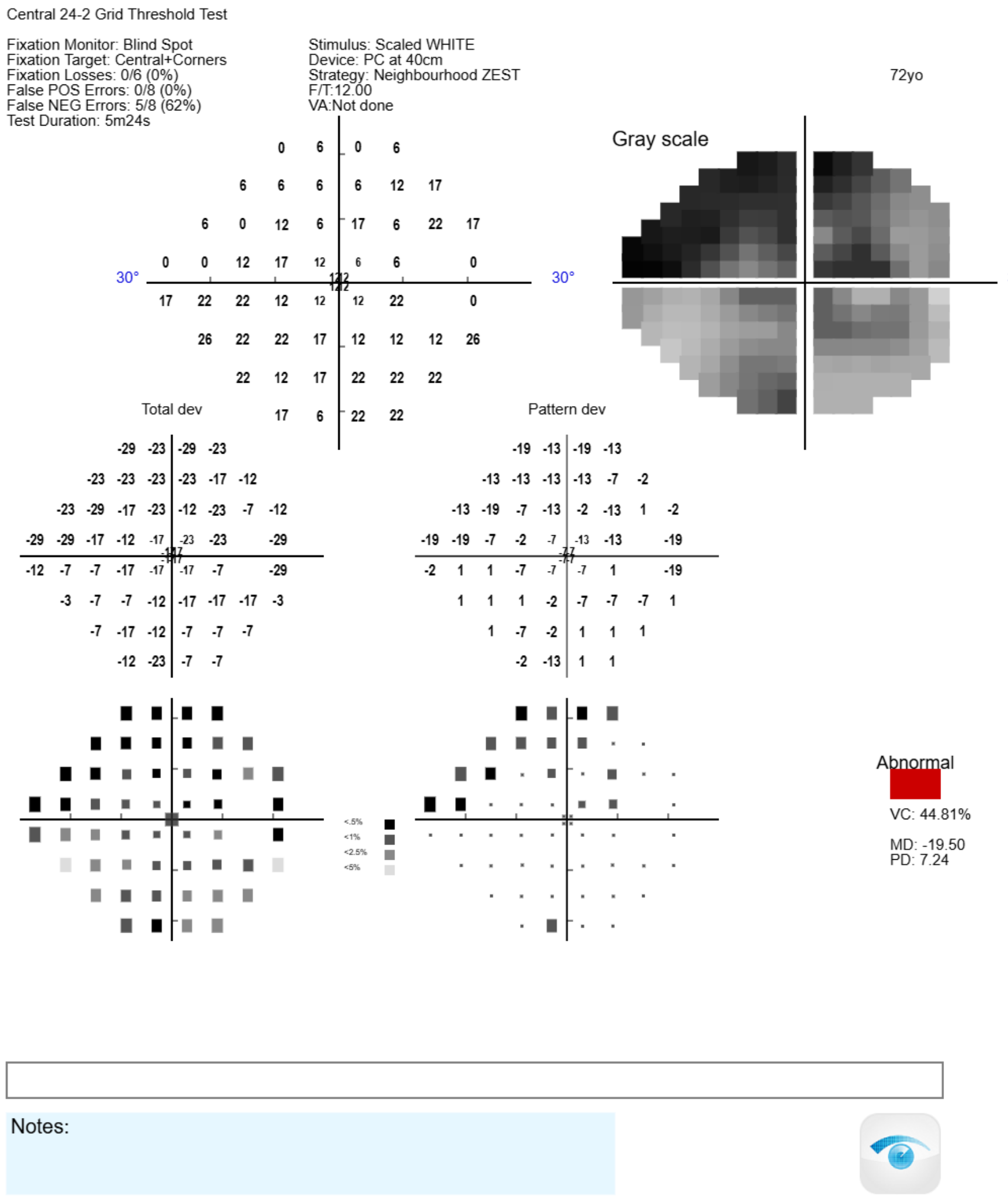

MRF Test 2

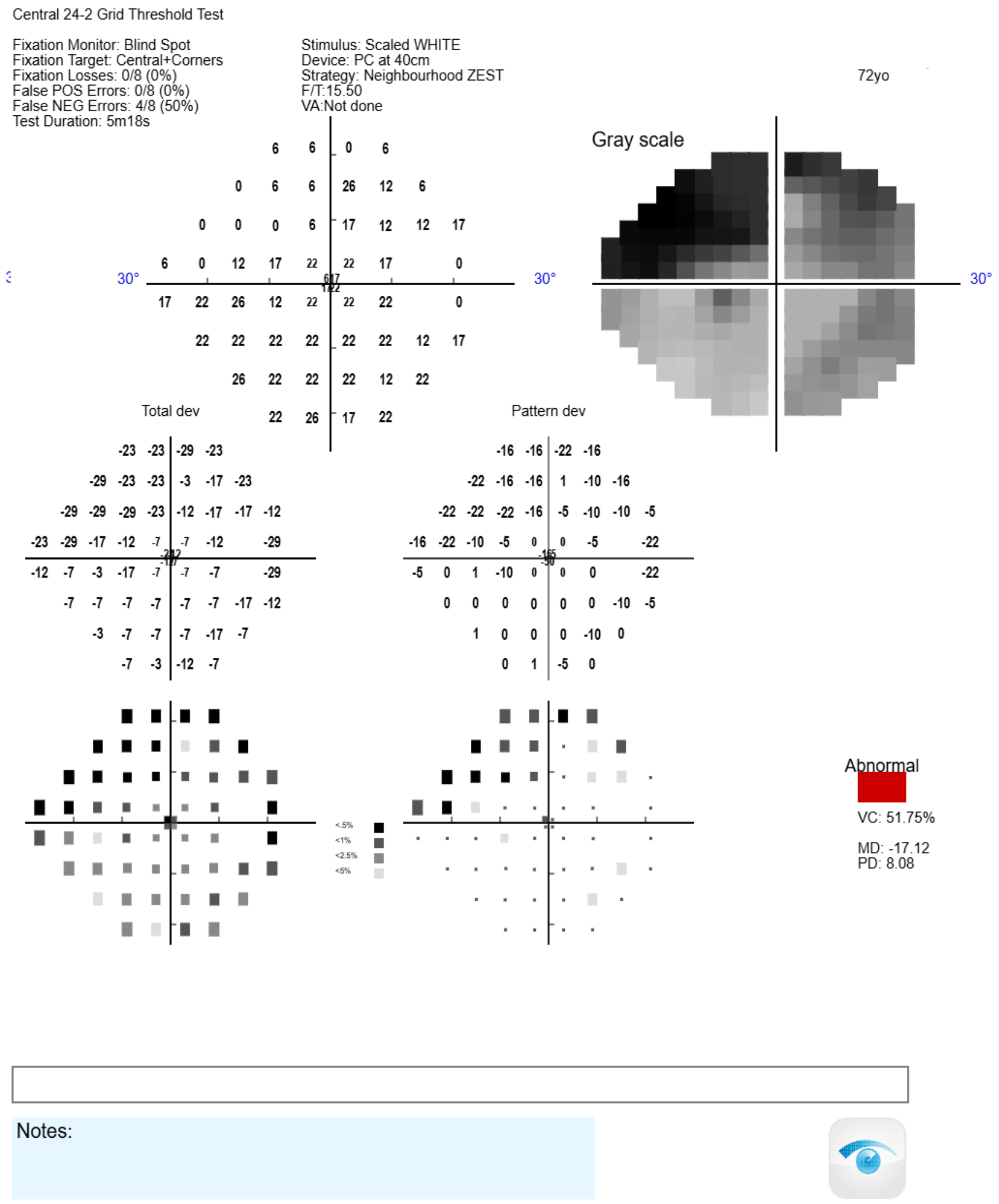

Example Octopus 600 vs MRF outputs

Severe Glaucoma Case 2

Octopus 600

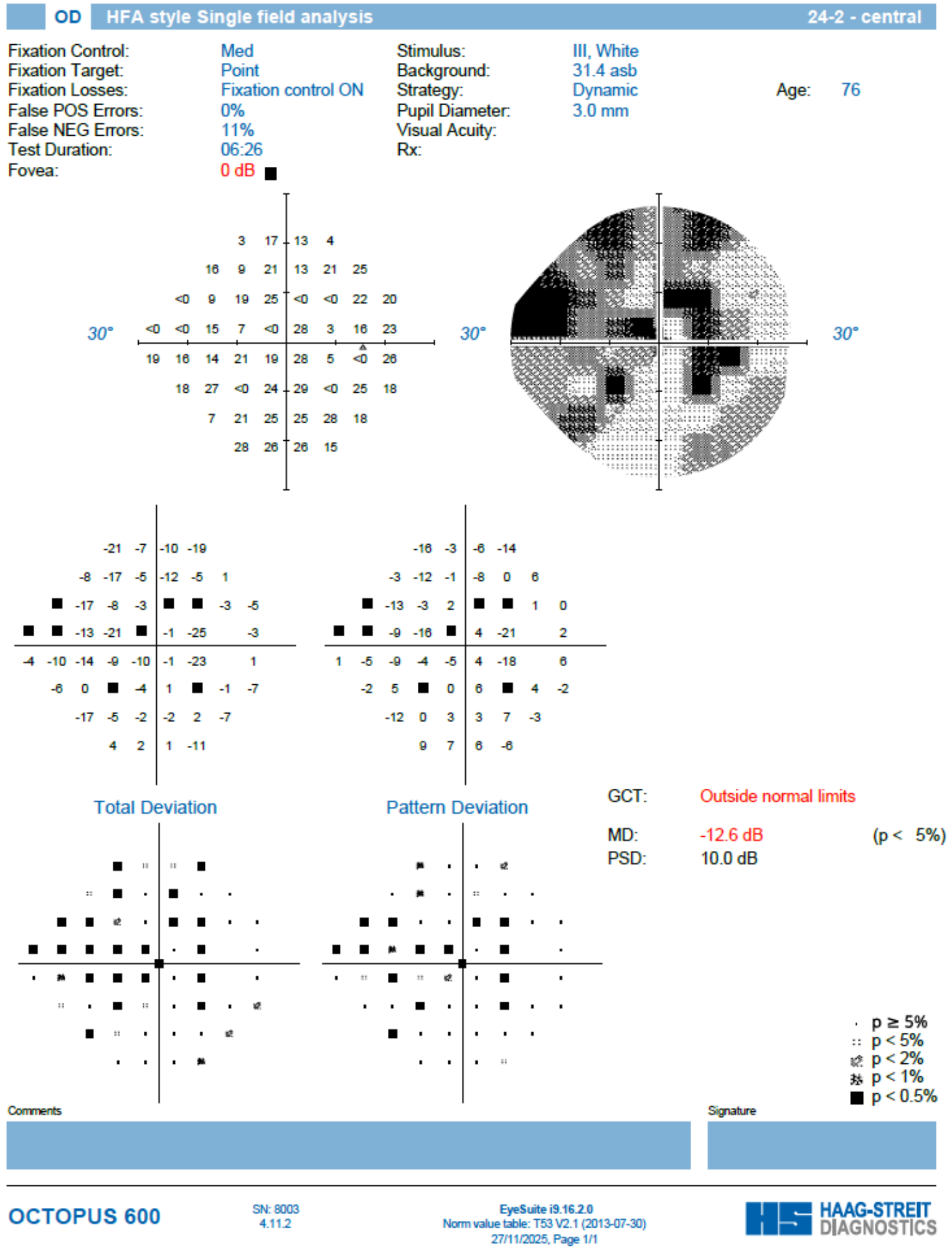

MRF Test 1

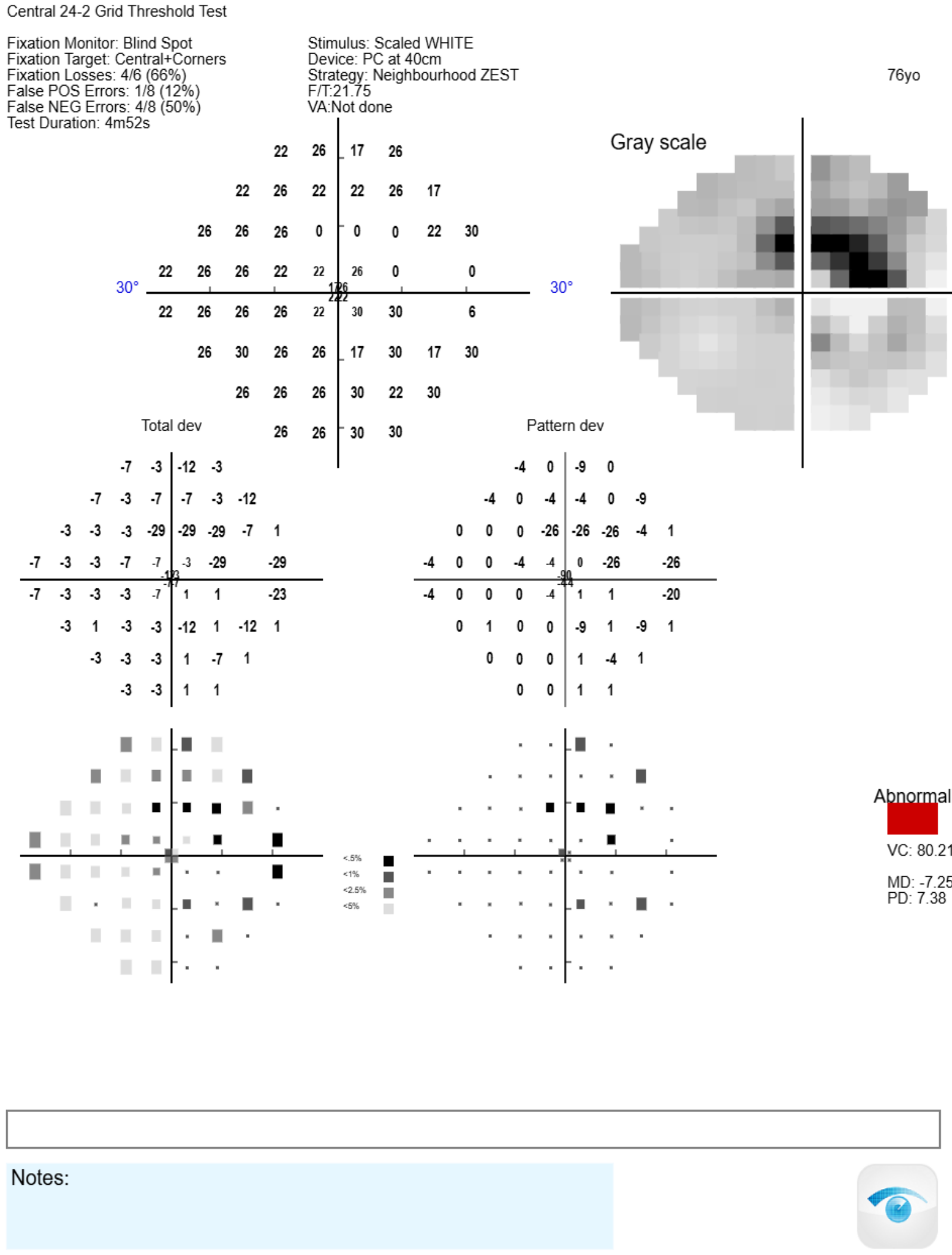

MRF Test 2

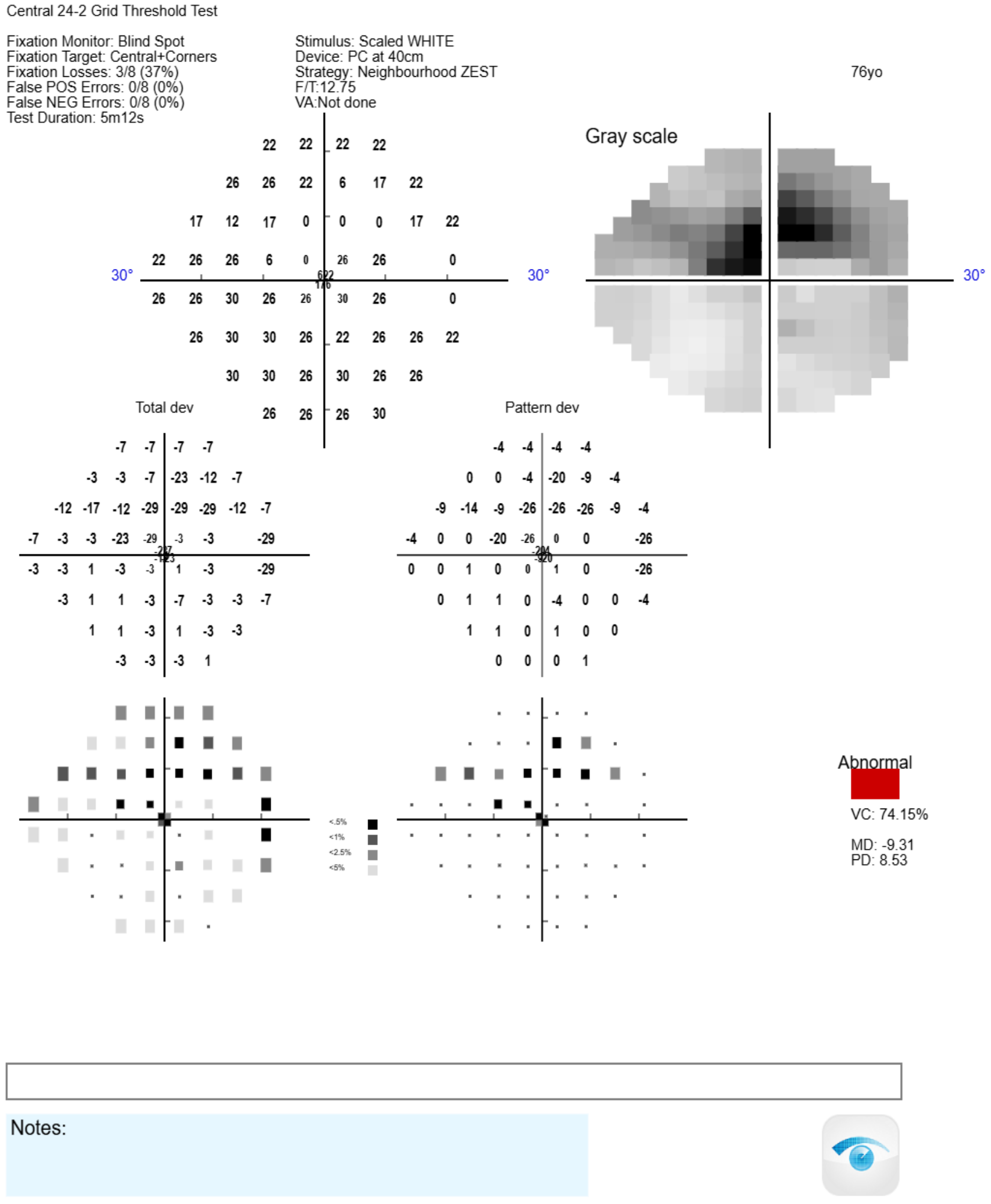

Example Octopus 600 vs MRF outputs

Severe Glaucoma Case 3

Octopus 600

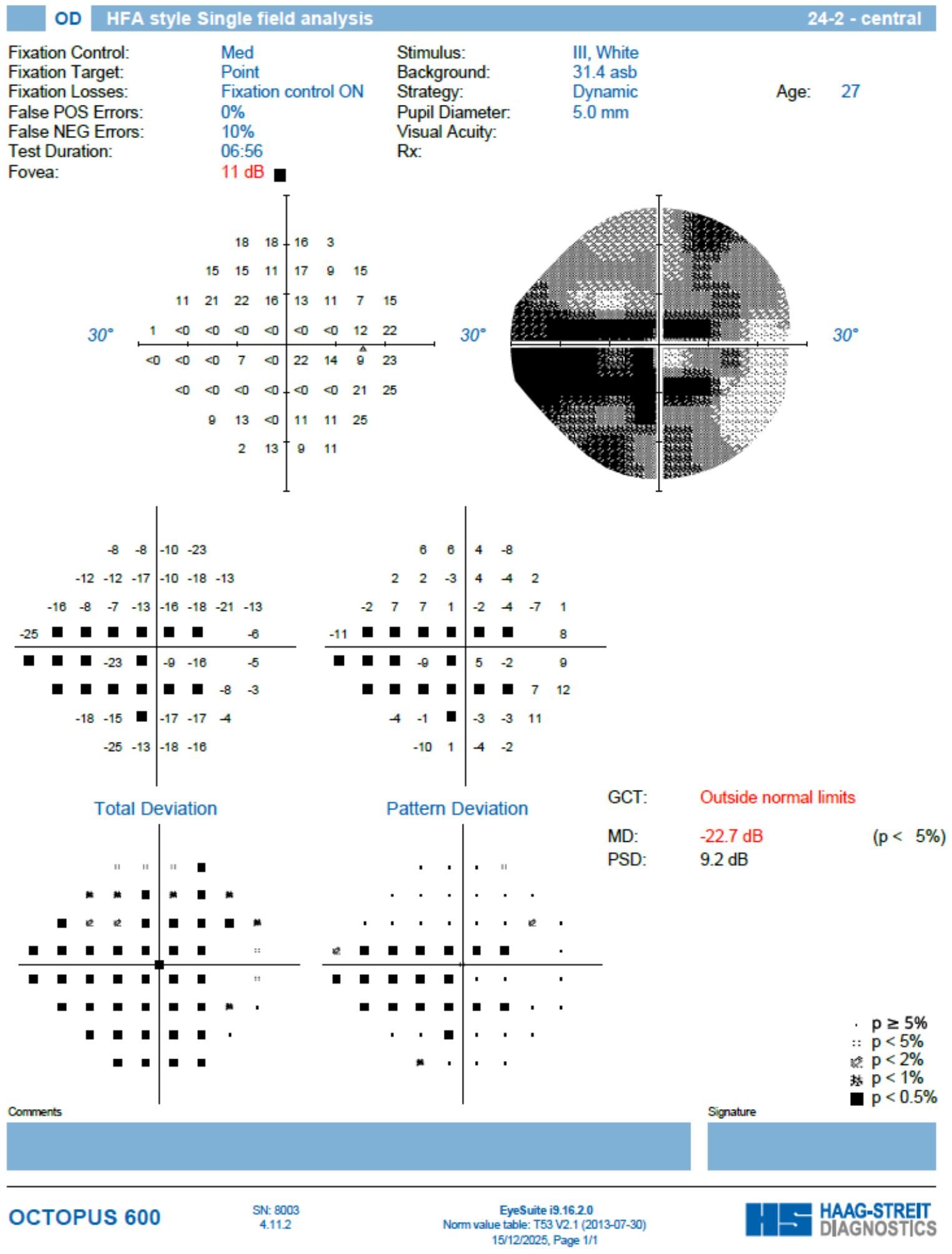

MRF Test 1

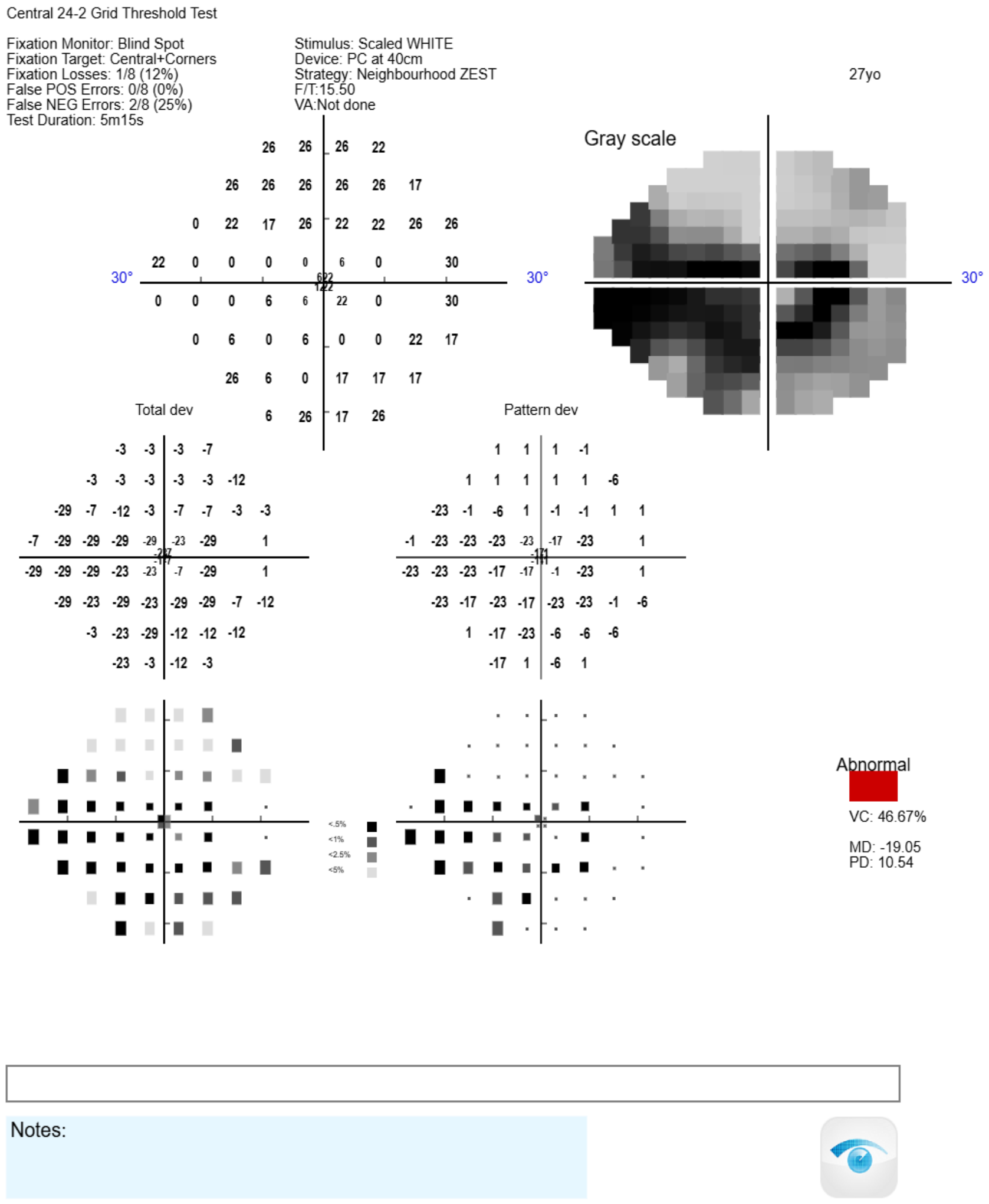

MRF Test 2

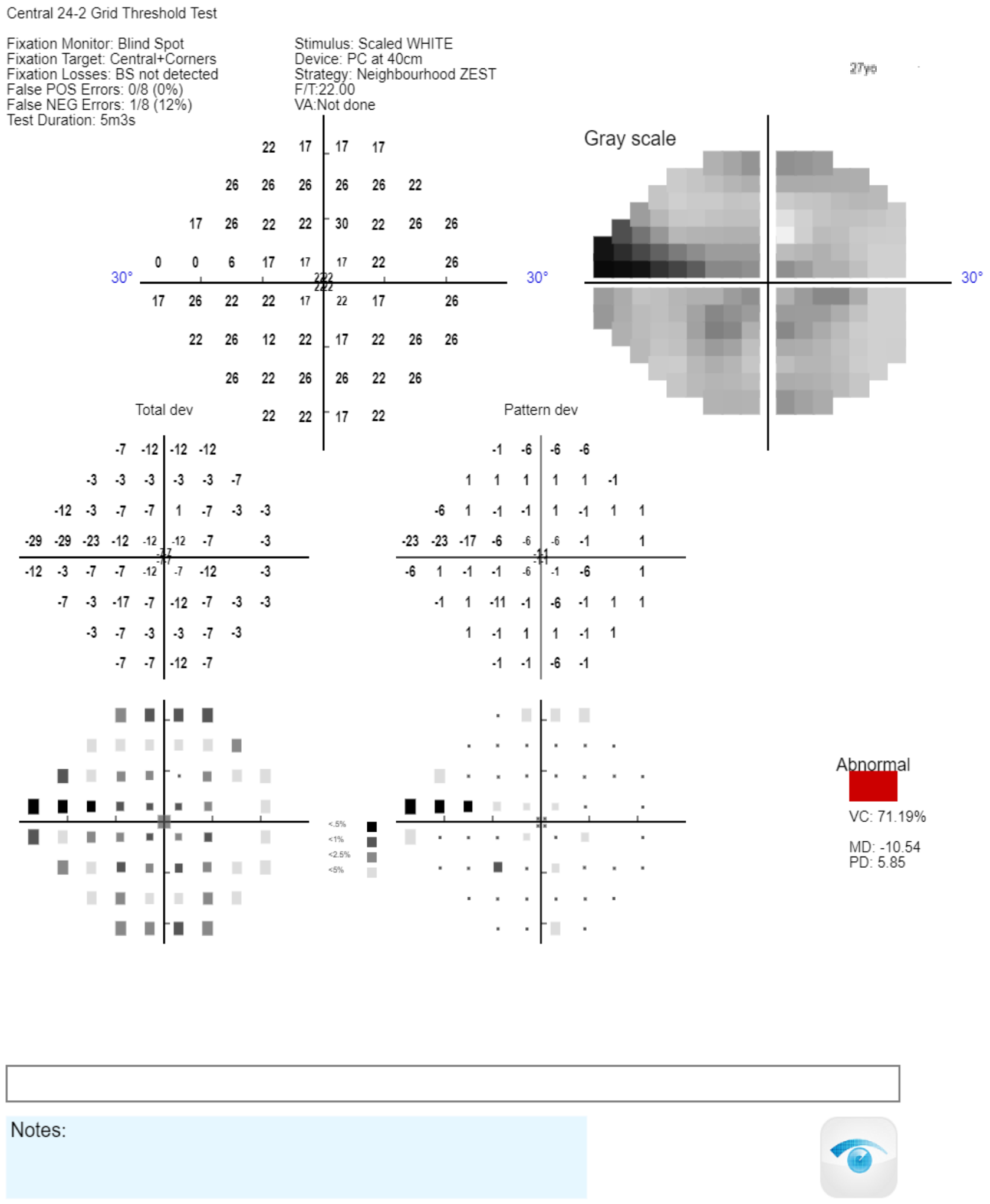

Example Octopus 600 vs MRF outputs

Severe Glaucoma Case 4

Octopus 600

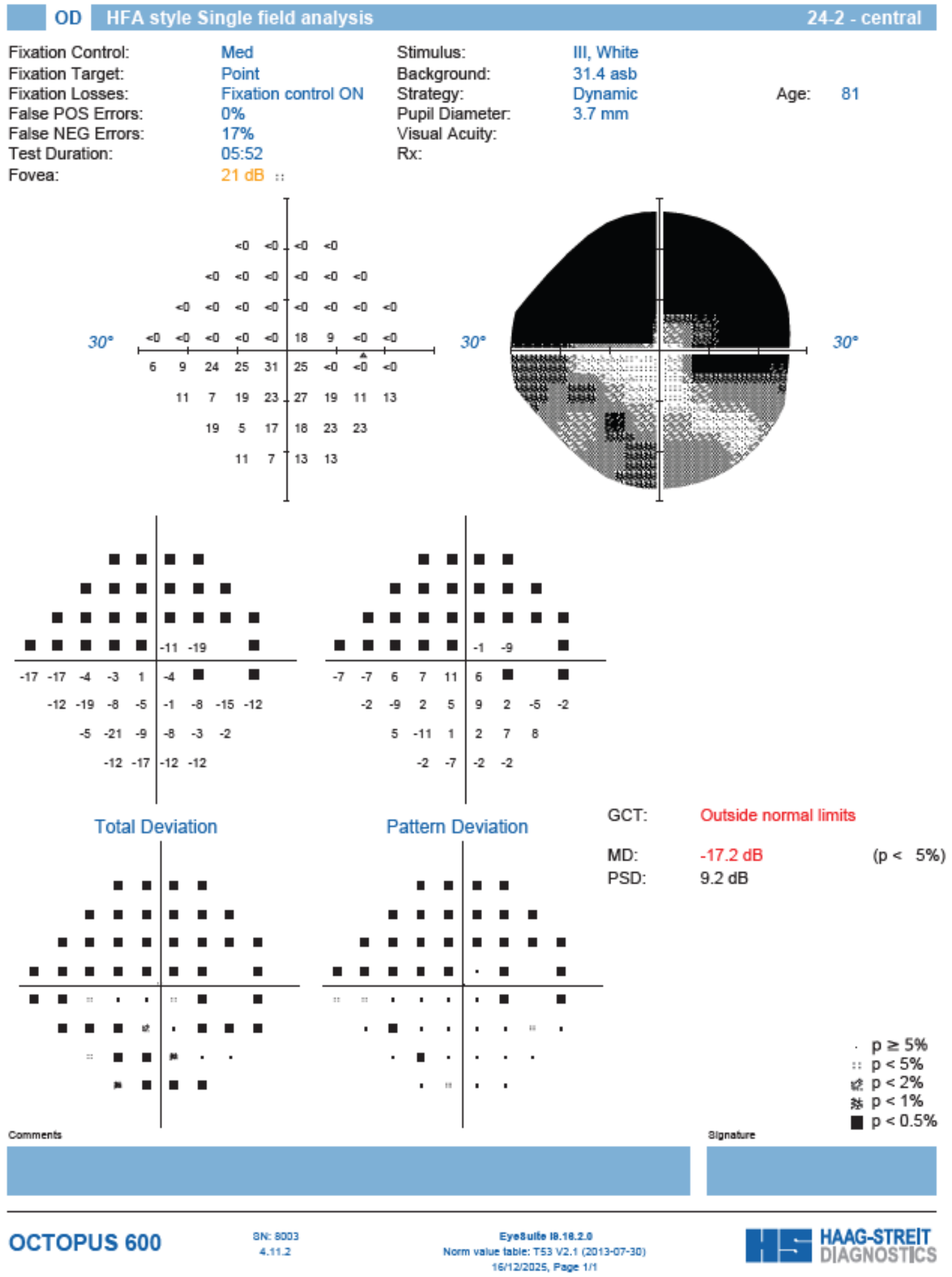

MRF Test 1

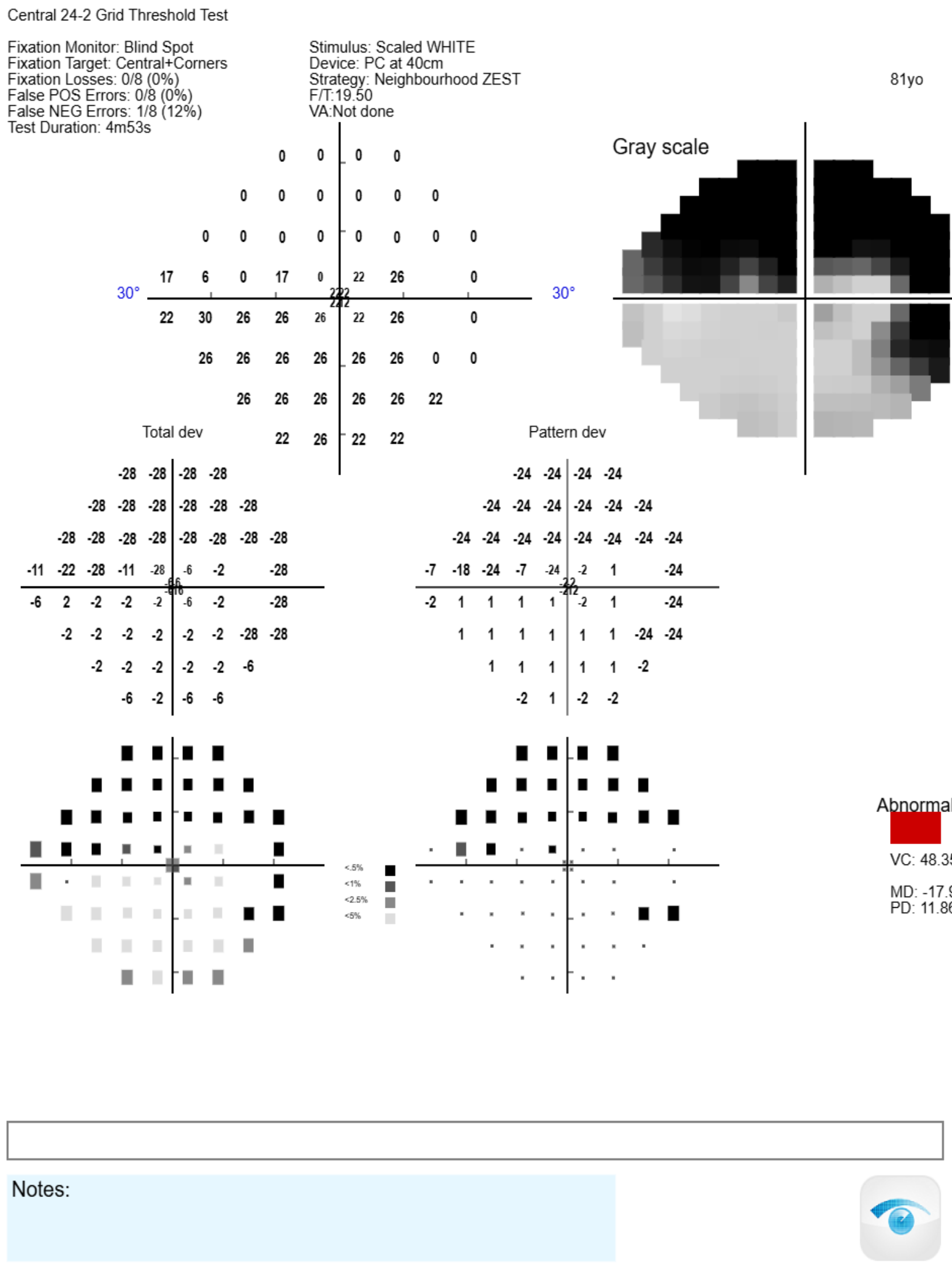

MRF Test 2

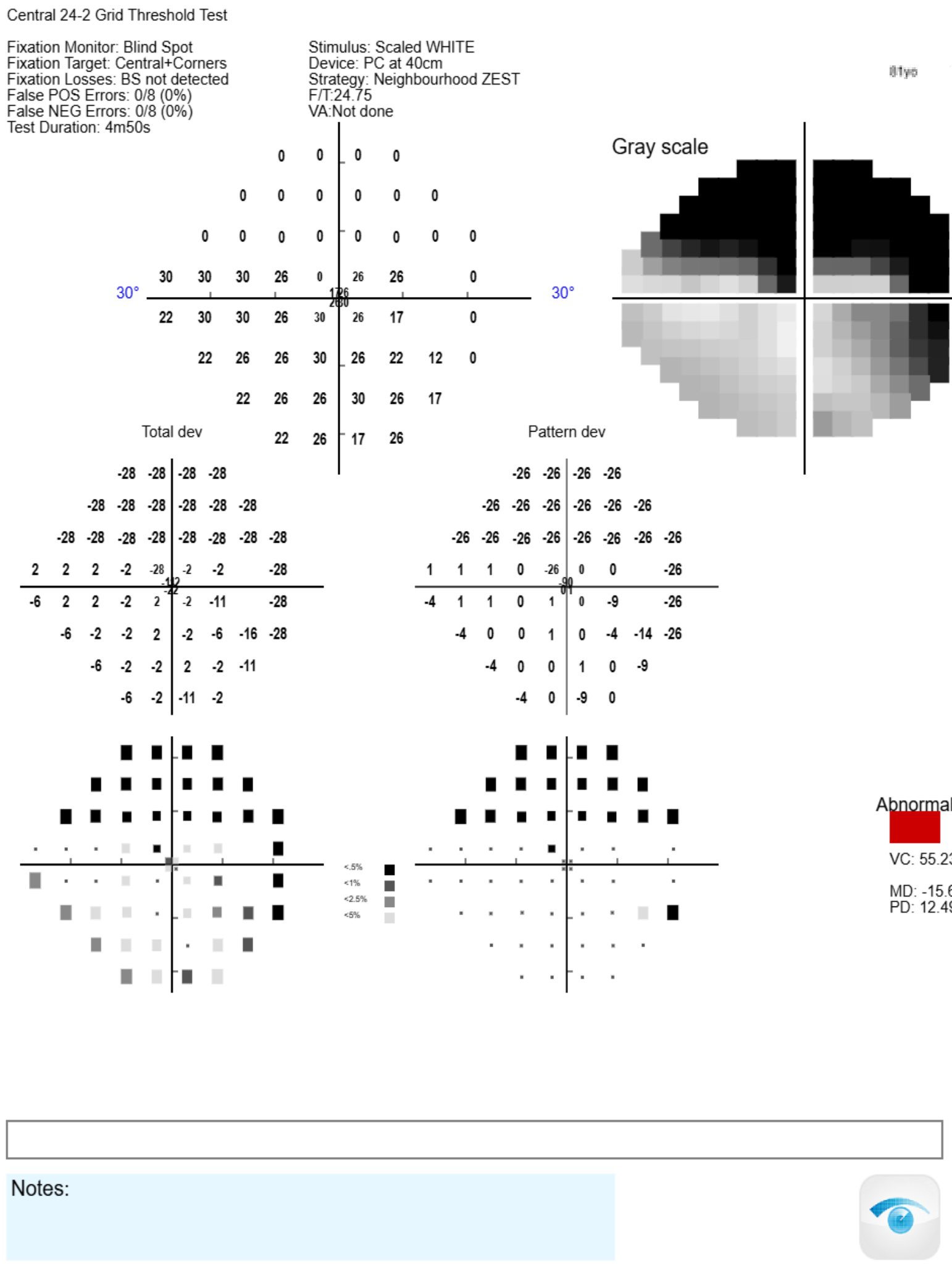

Example Octopus 600 vs MRF outputs

Moderate Glaucoma Case 1

Octopus 600

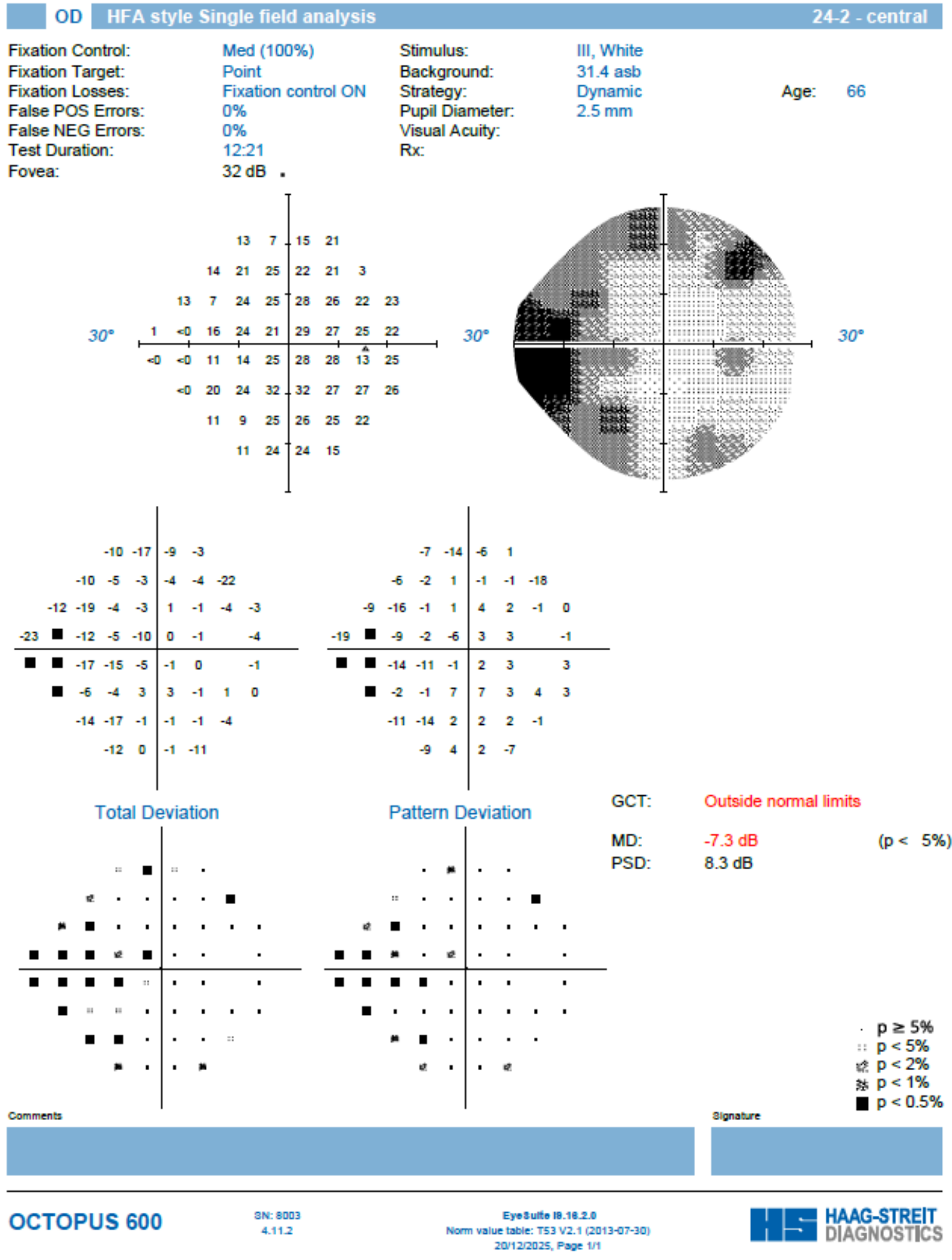

MRF Test 1

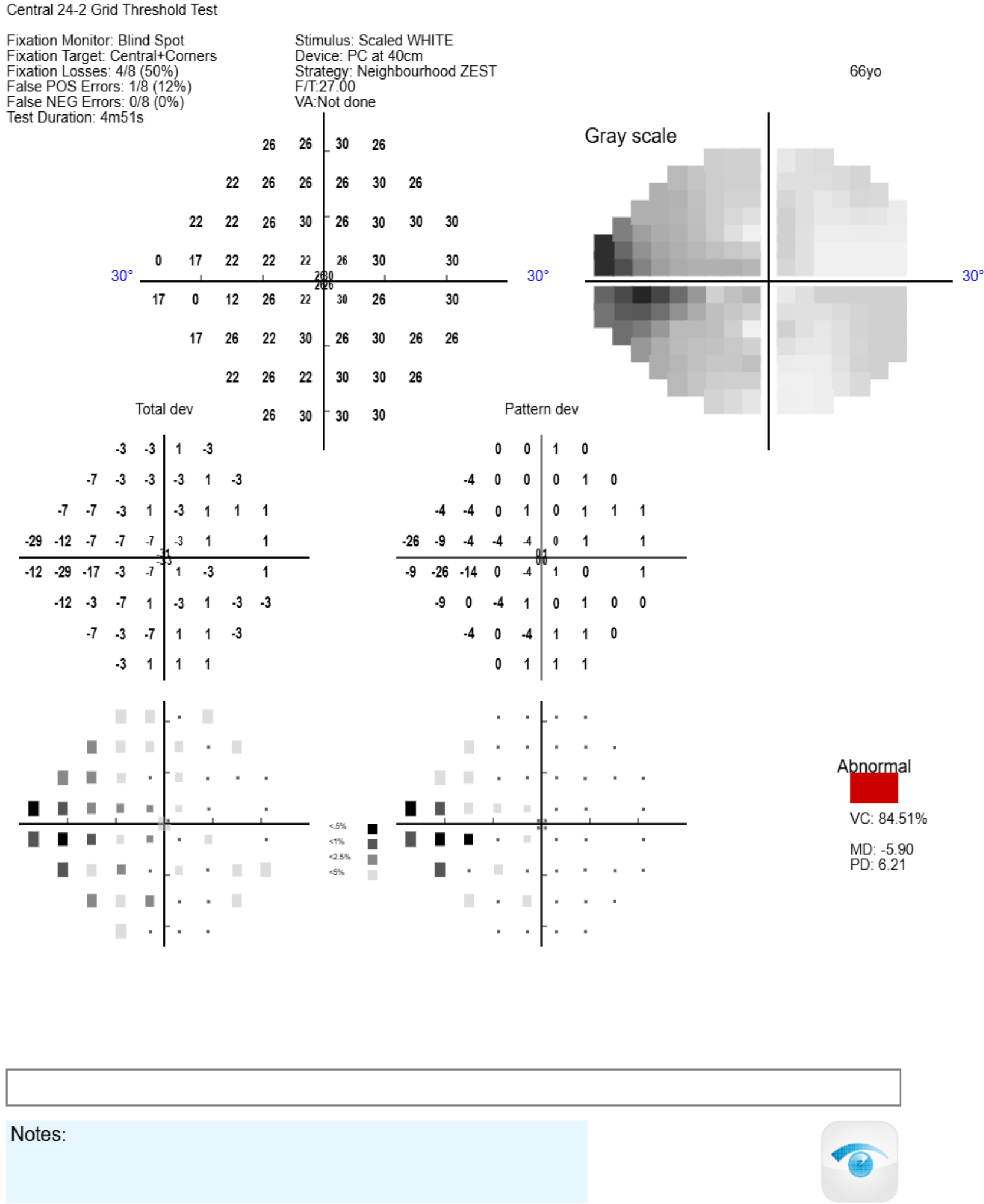

MRF Test 2

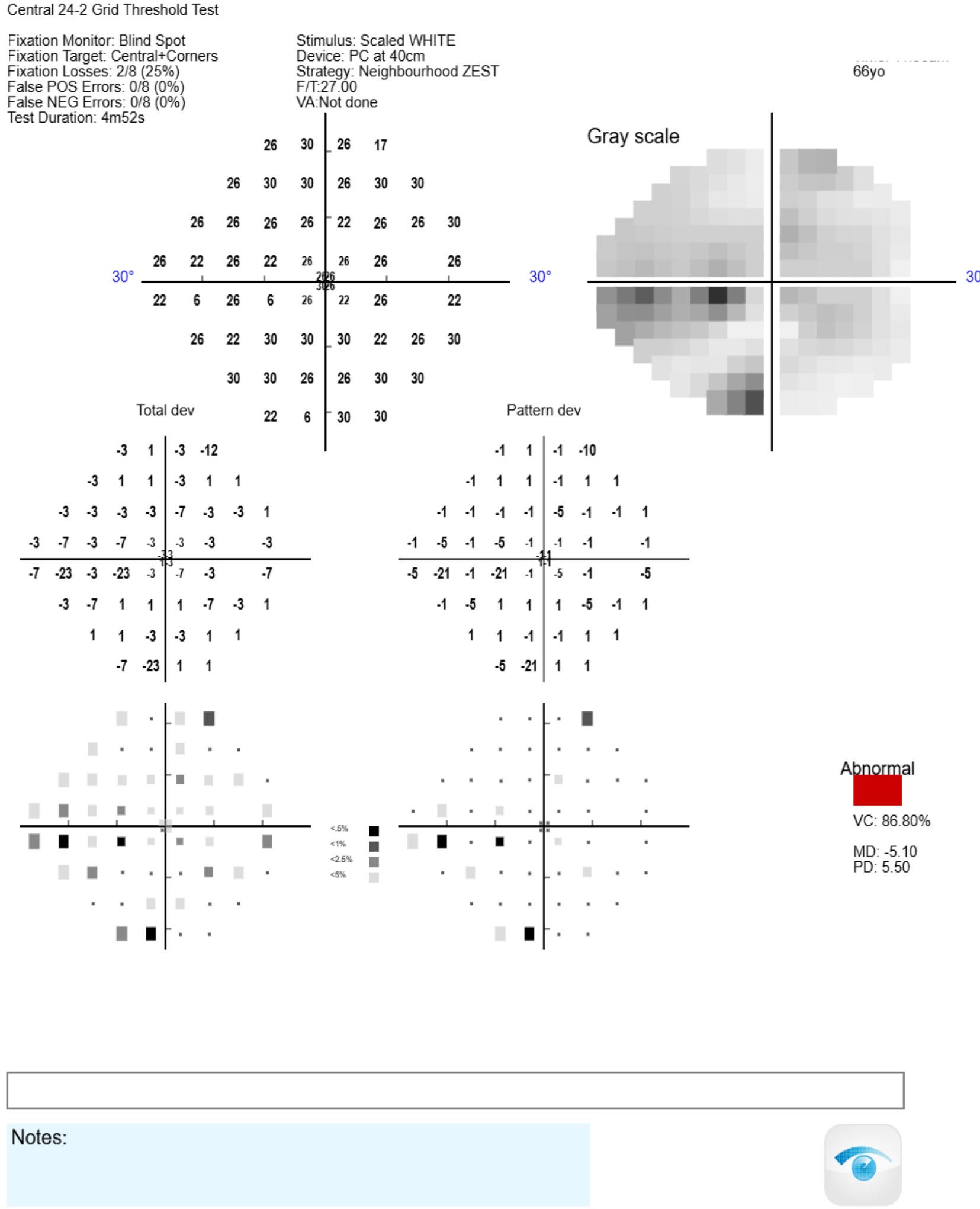

Example Octopus 600 vs MRF outputs

Moderate Glaucoma Case 2

Octopus 600

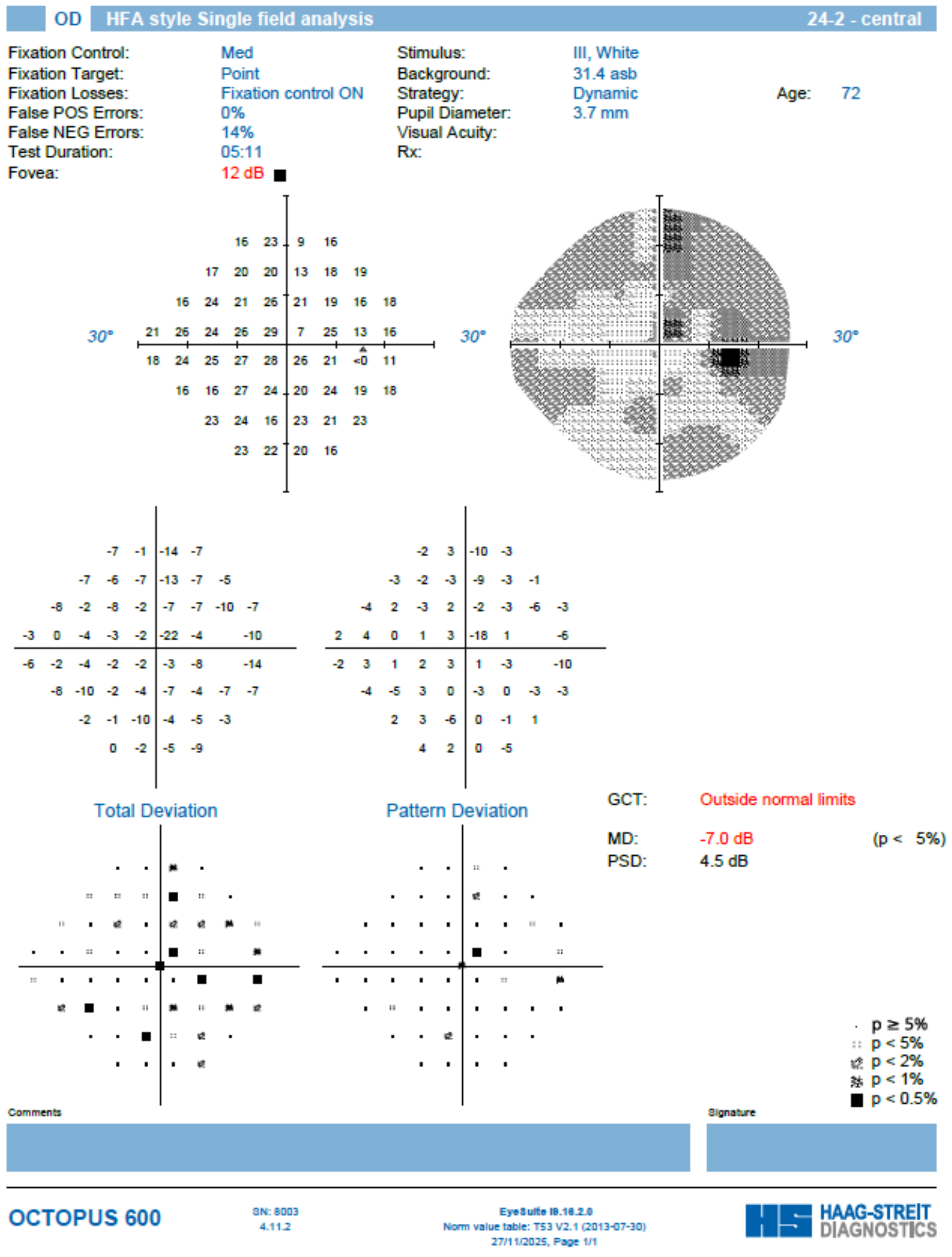

MRF Test 1

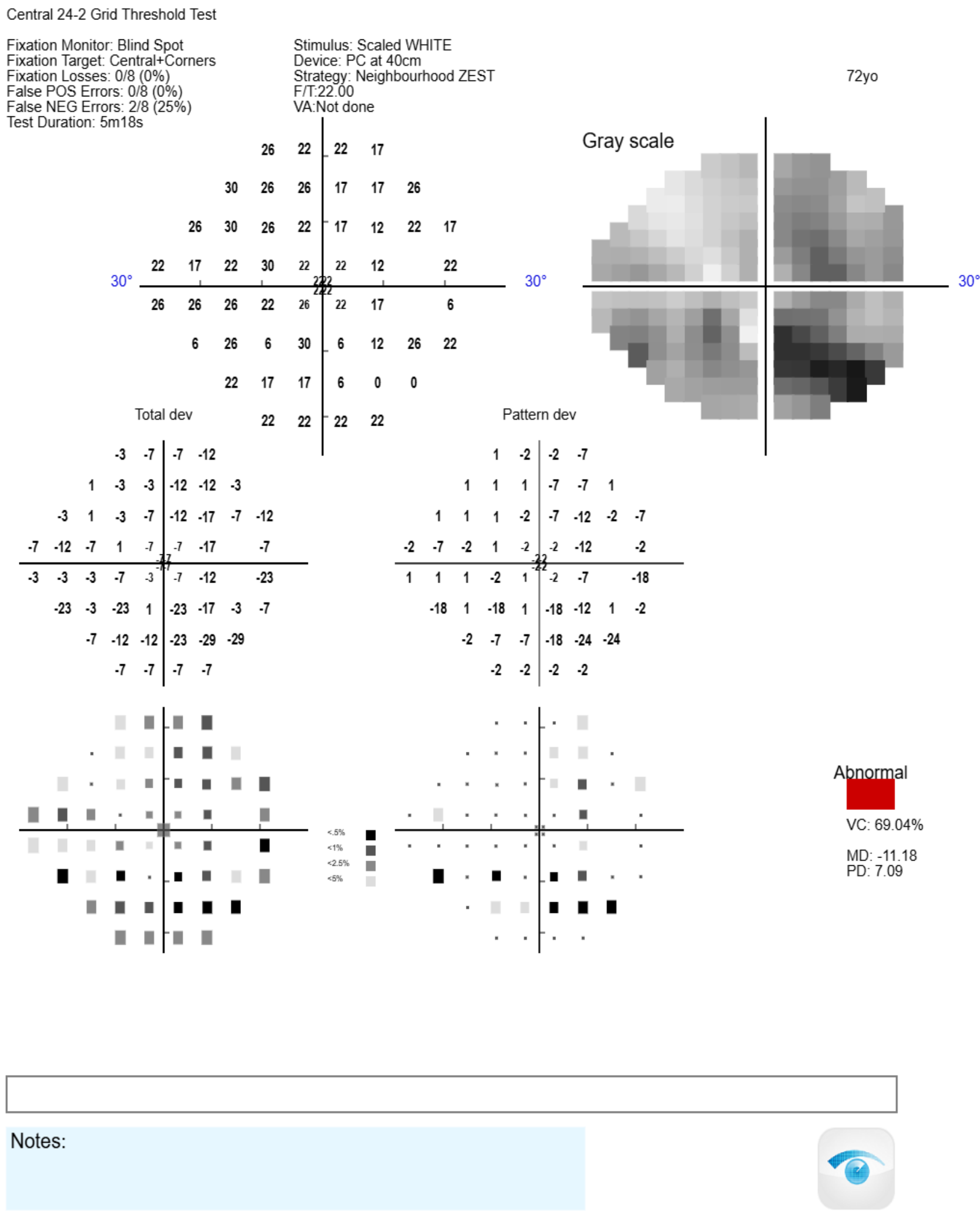

MRF Test 2

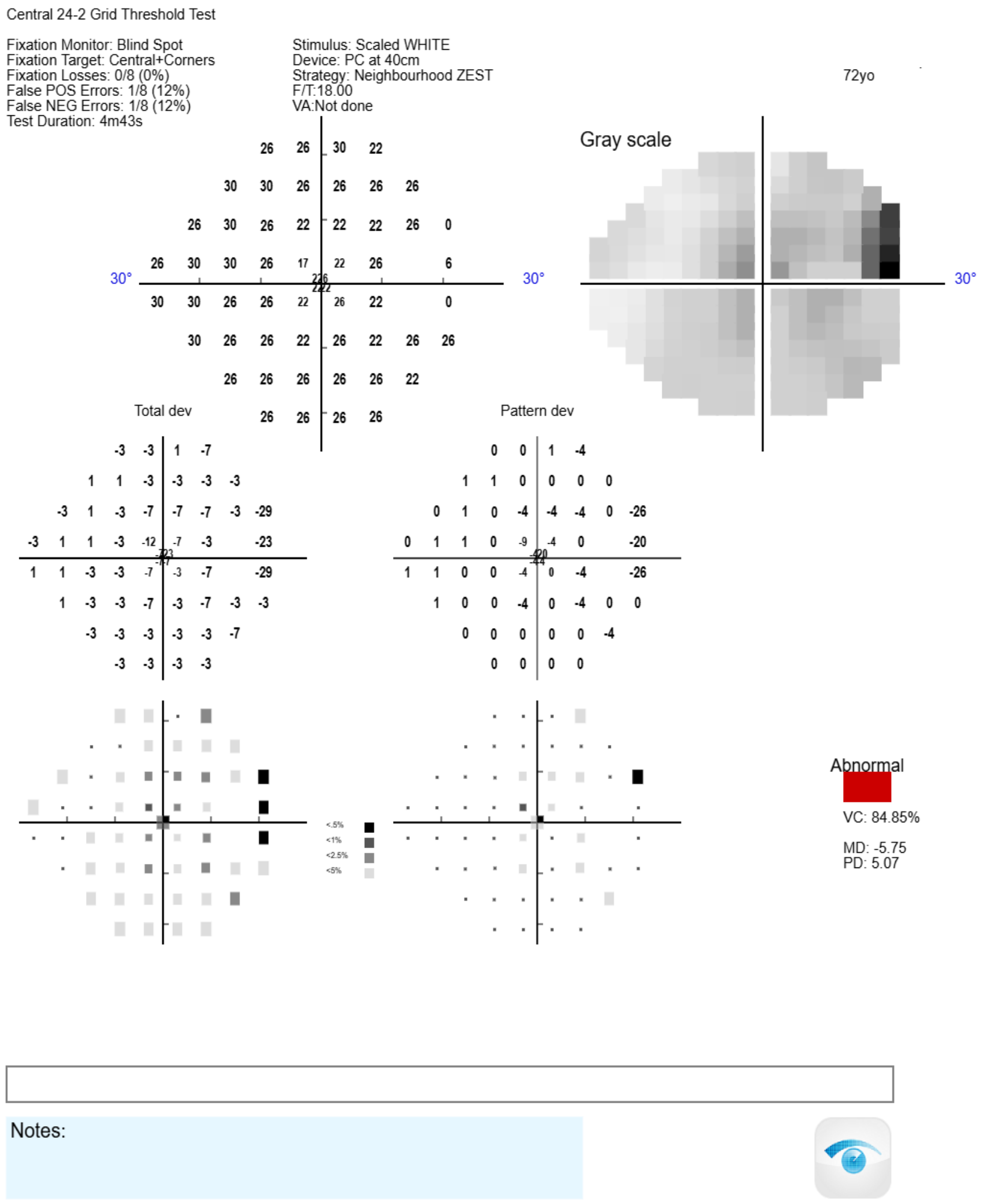

Example Octopus 600 vs MRF outputs

Moderate Glaucoma Case 3

Octopus 600

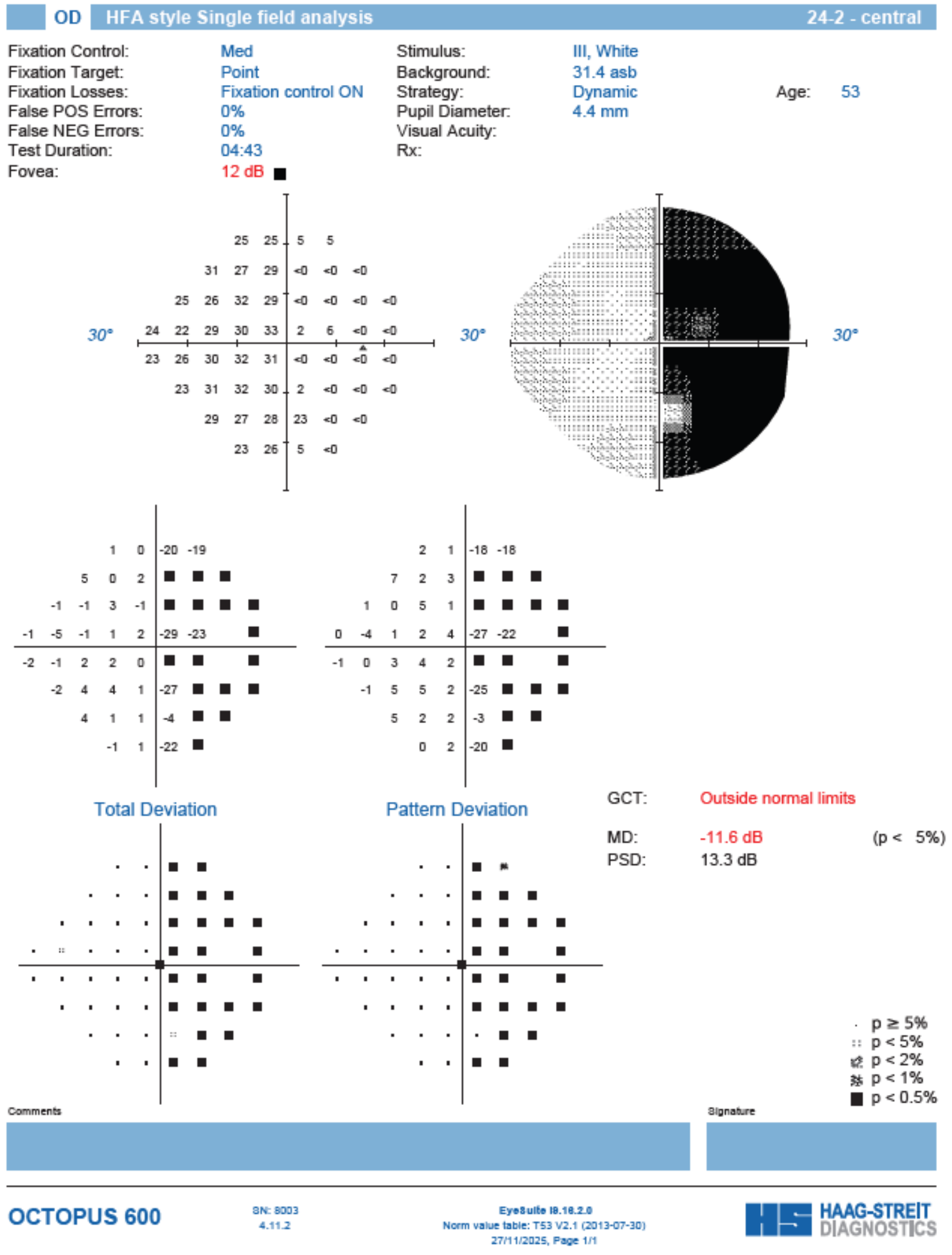

MRF Test 1

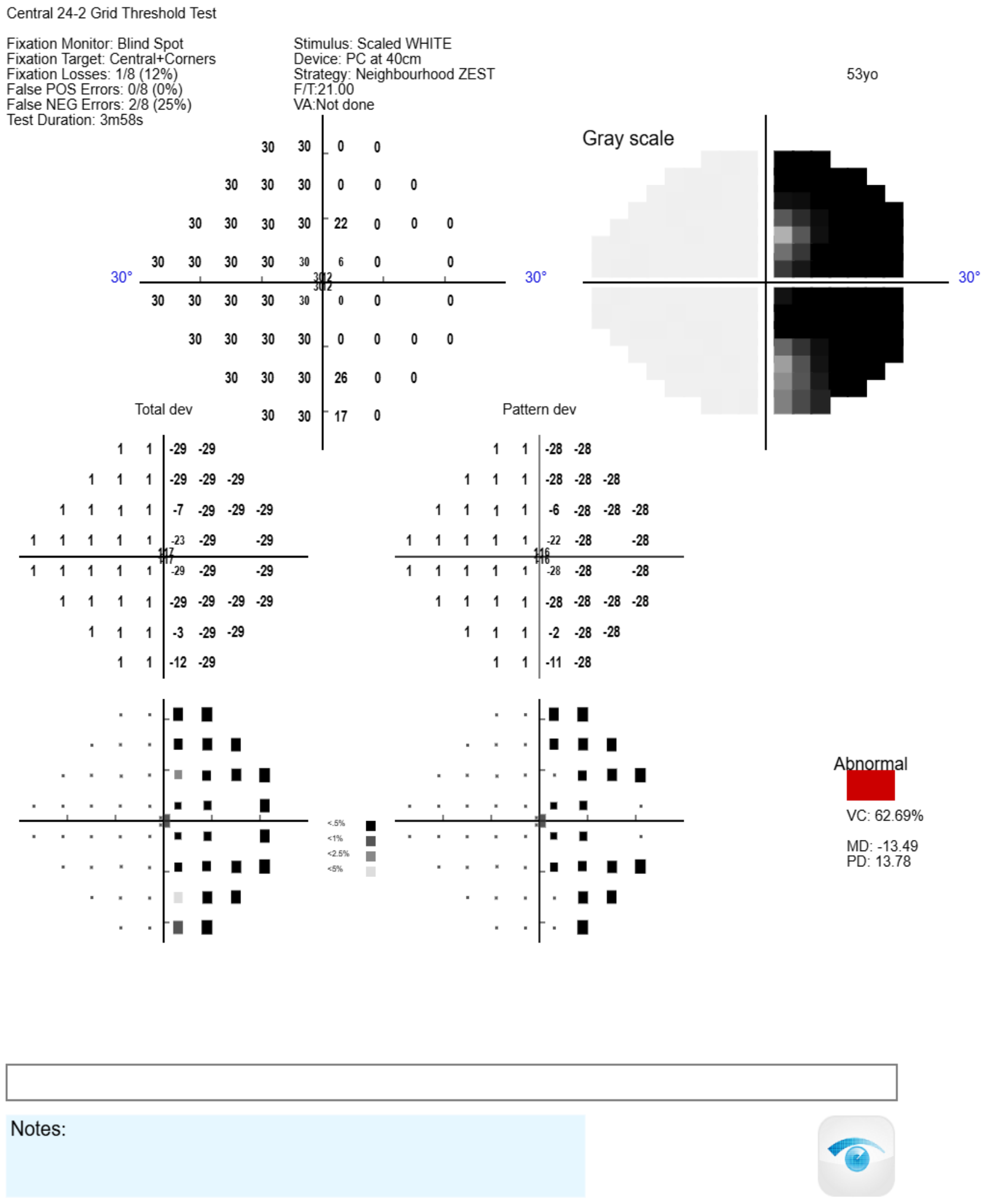

MRF Test 2

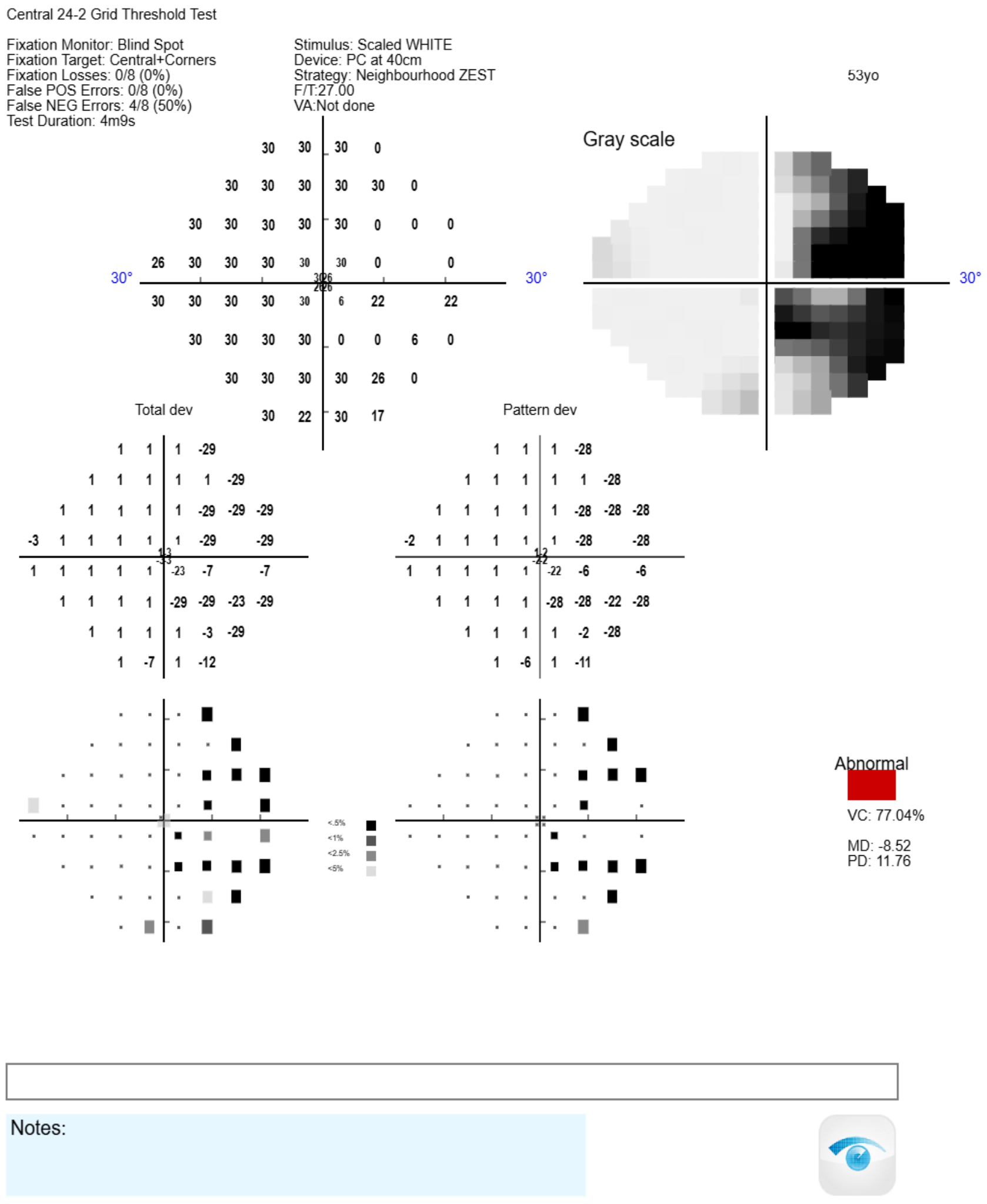

Example Octopus 600 vs MRF outputs

Mild Glaucoma Case 1

Octopus 600

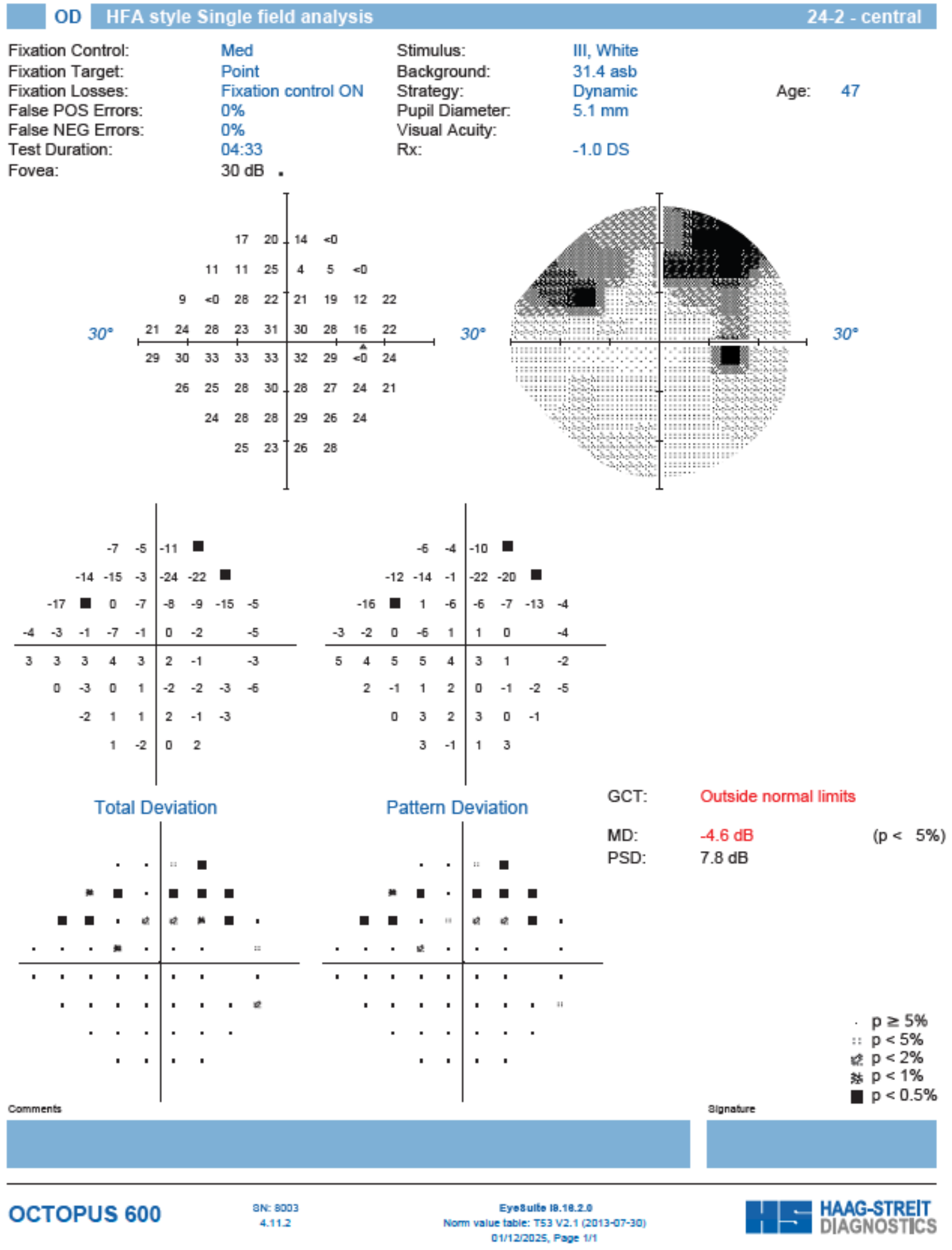

MRF Test 1

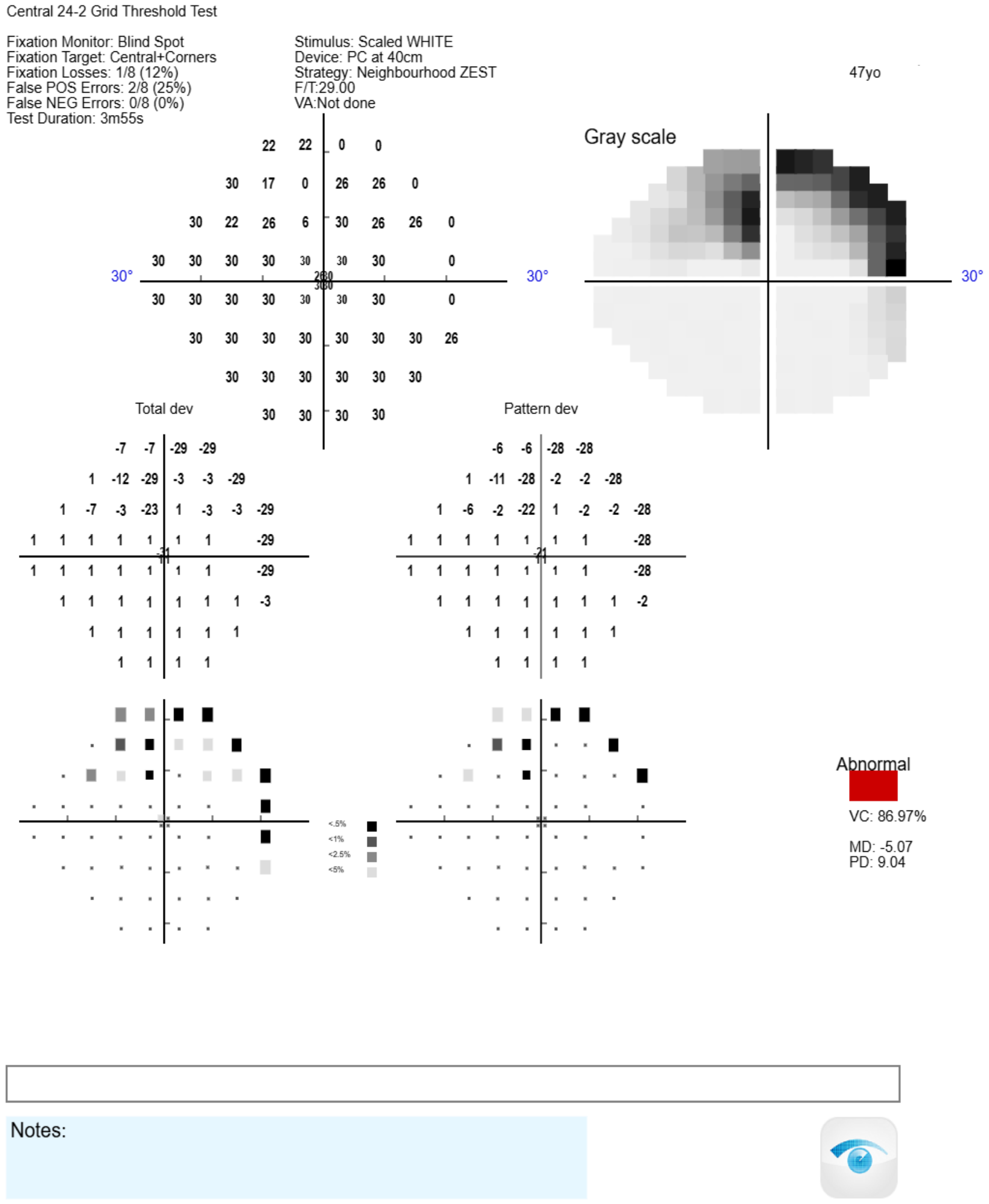

MRF Test 2

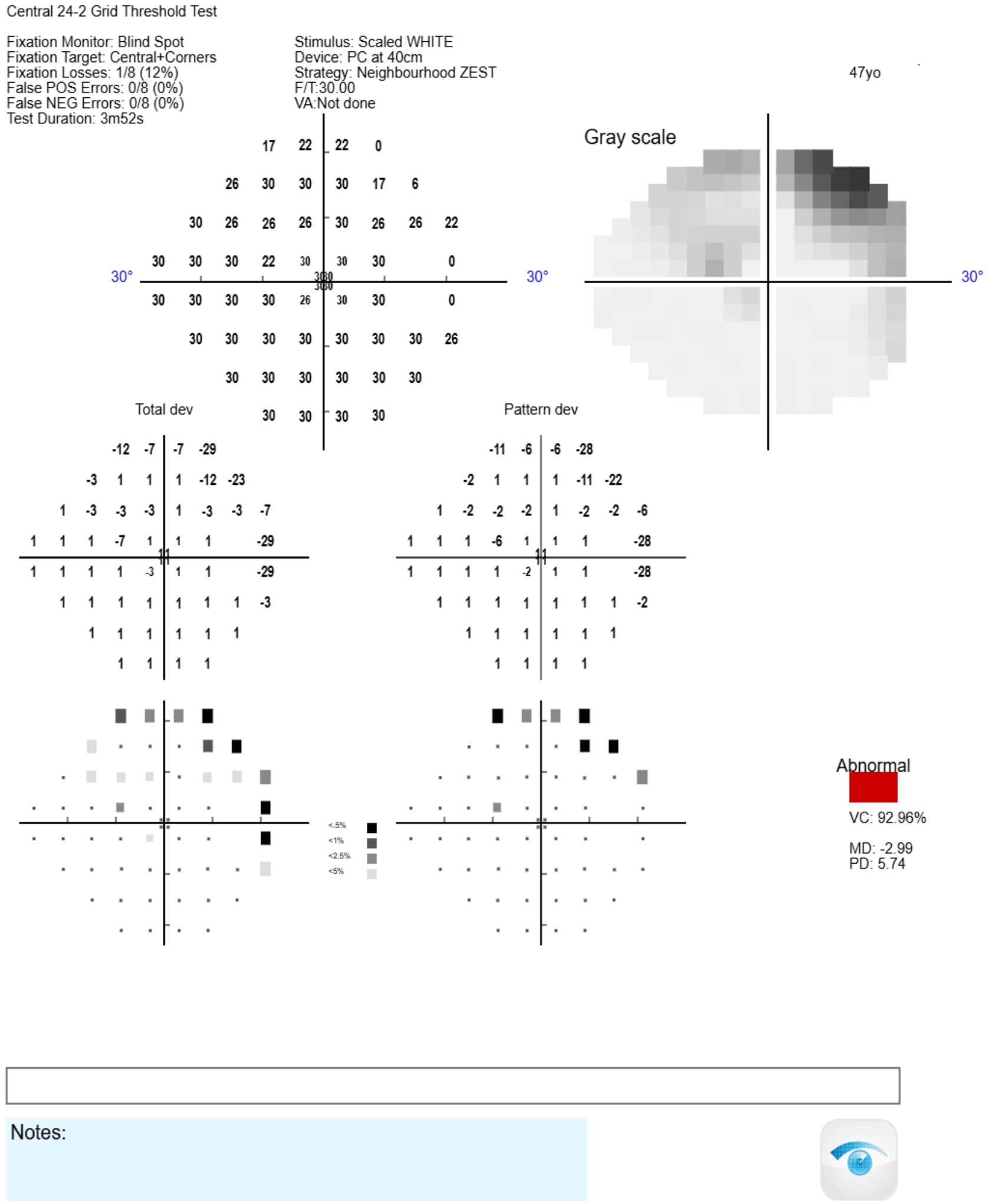

Example Octopus 600 vs MRF outputs

Mild Glaucoma Case 2

Octopus 600

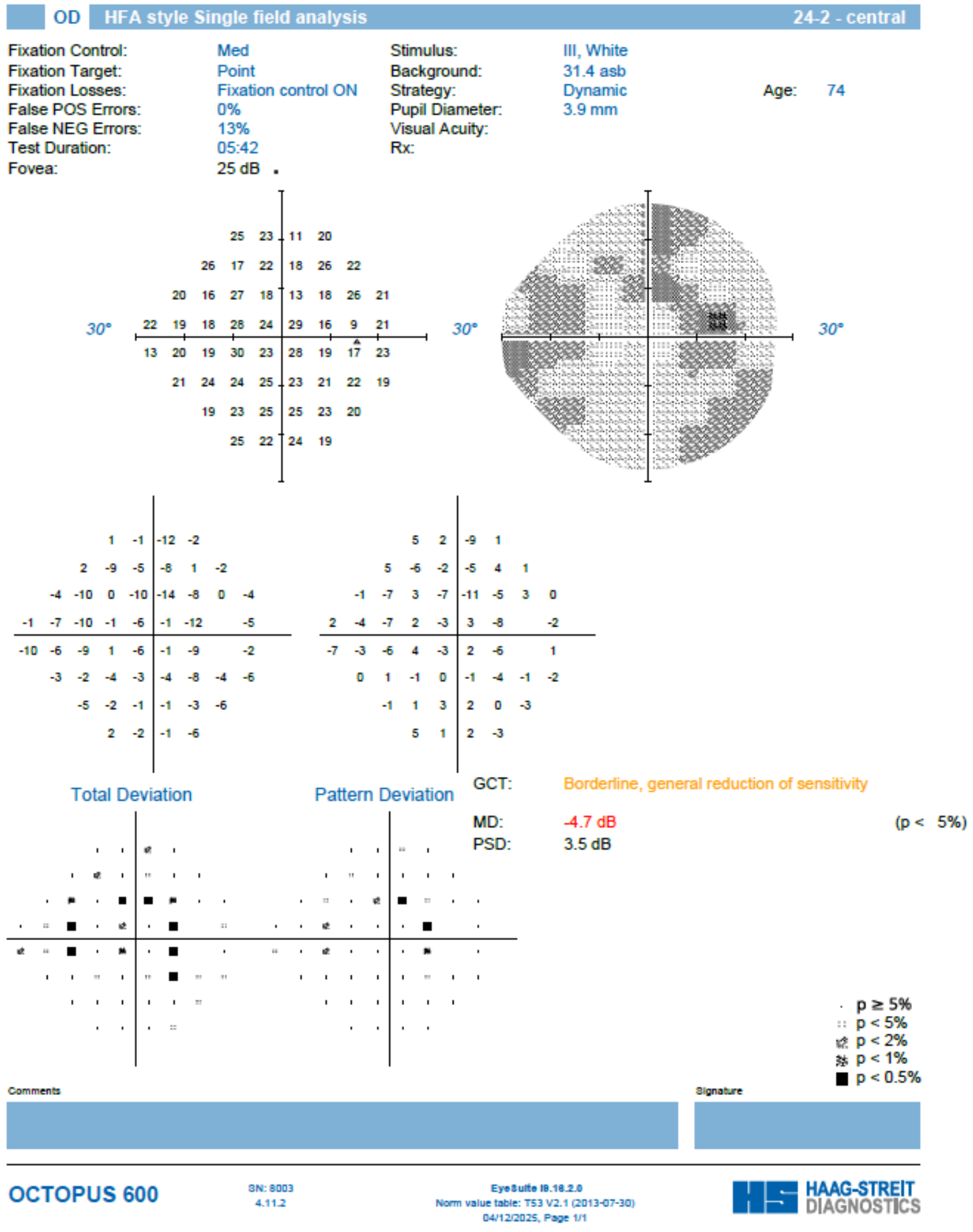

MRF Test 1

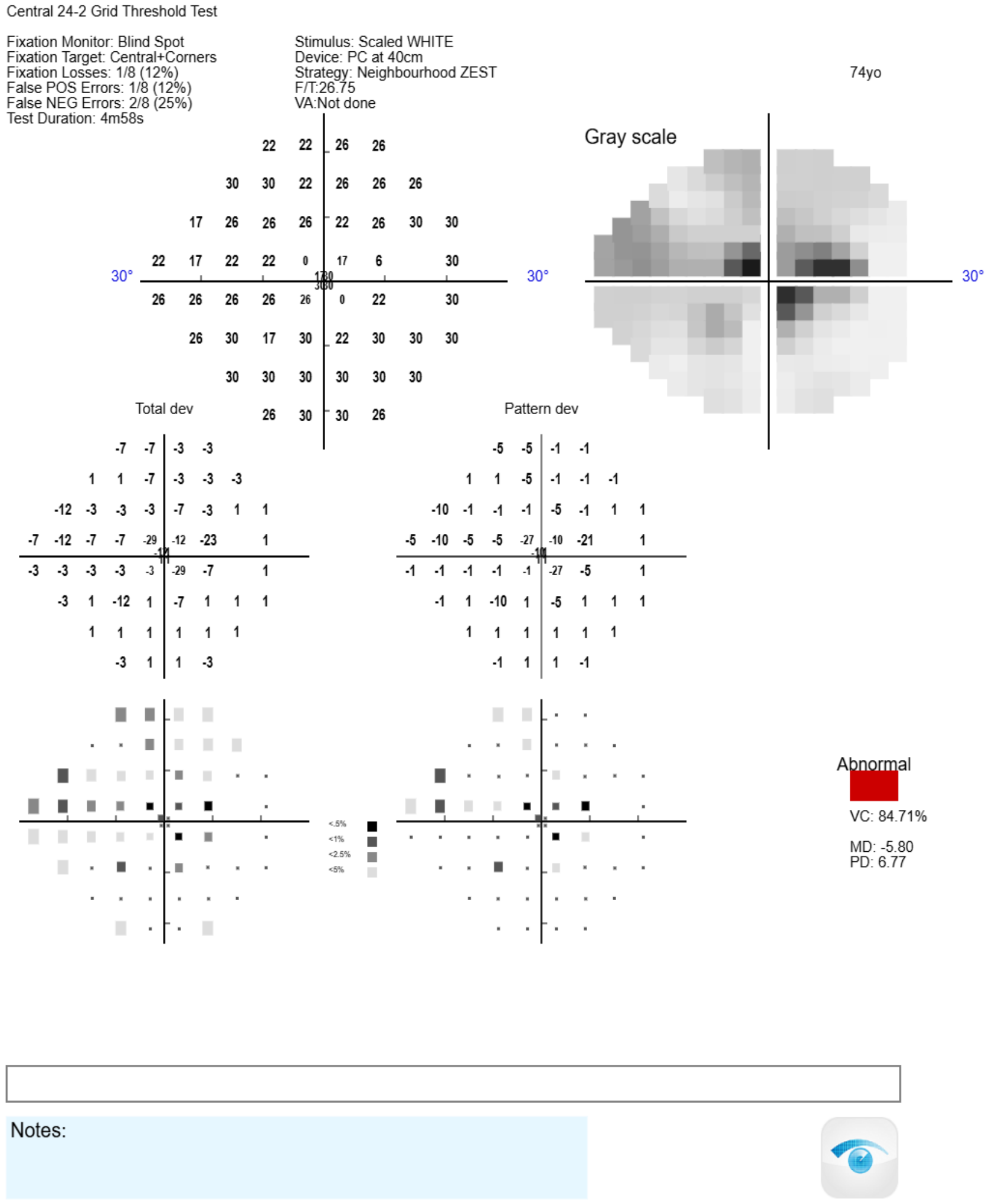

MRF Test 2

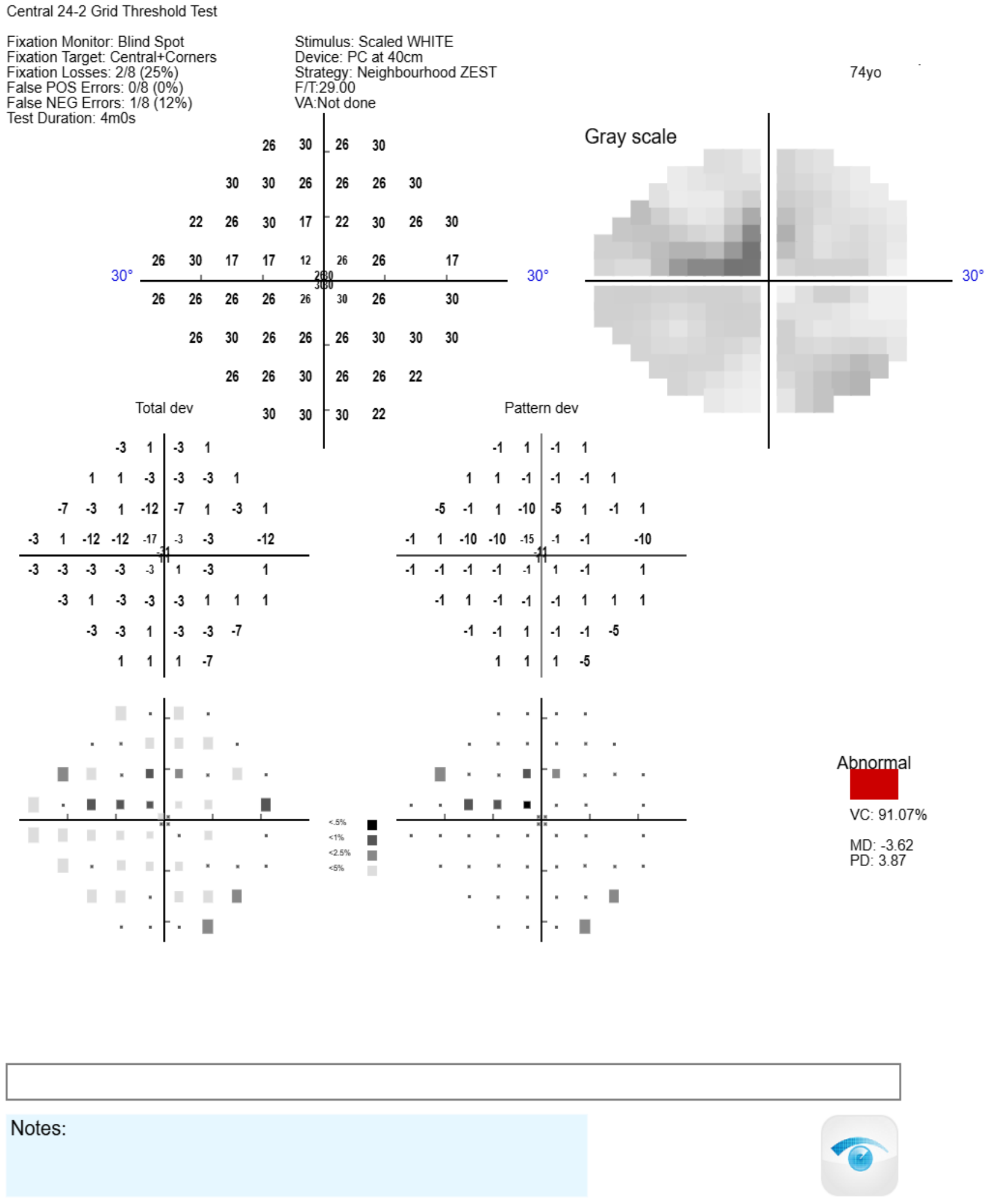

Example Octopus 600 vs MRF outputs

Mild Glaucoma Case 3

Octopus 600

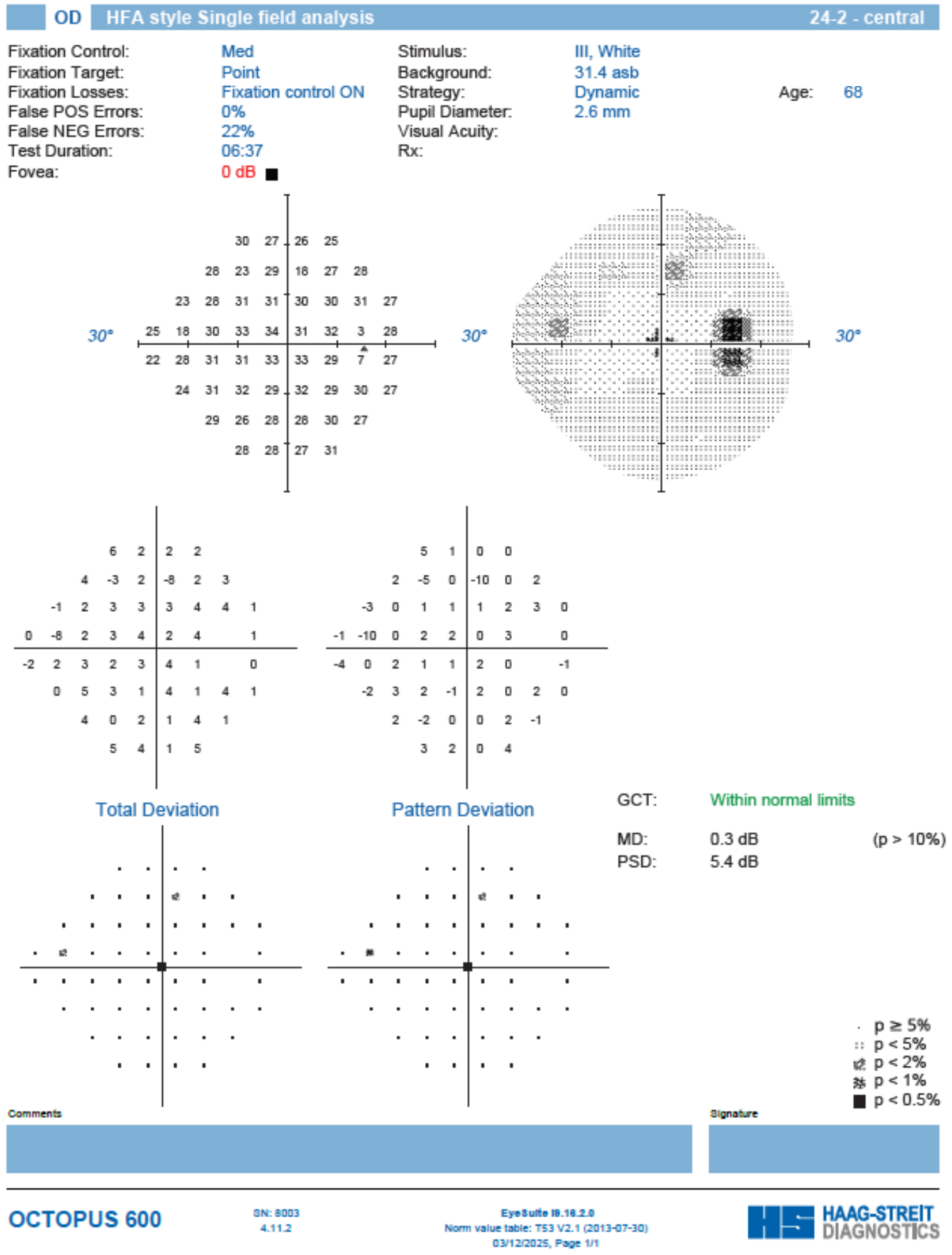

MRF Test 1

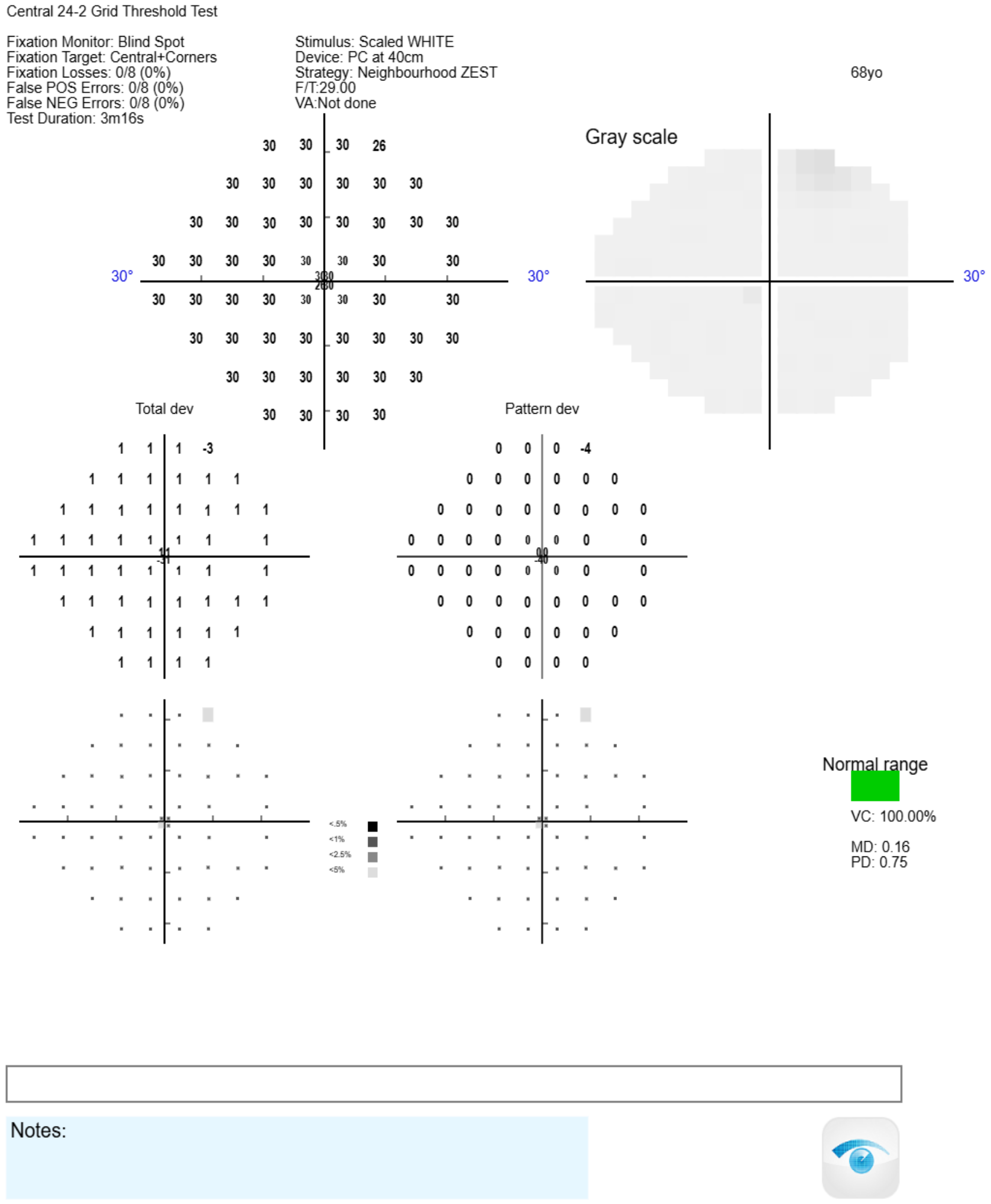

MRF Test 2

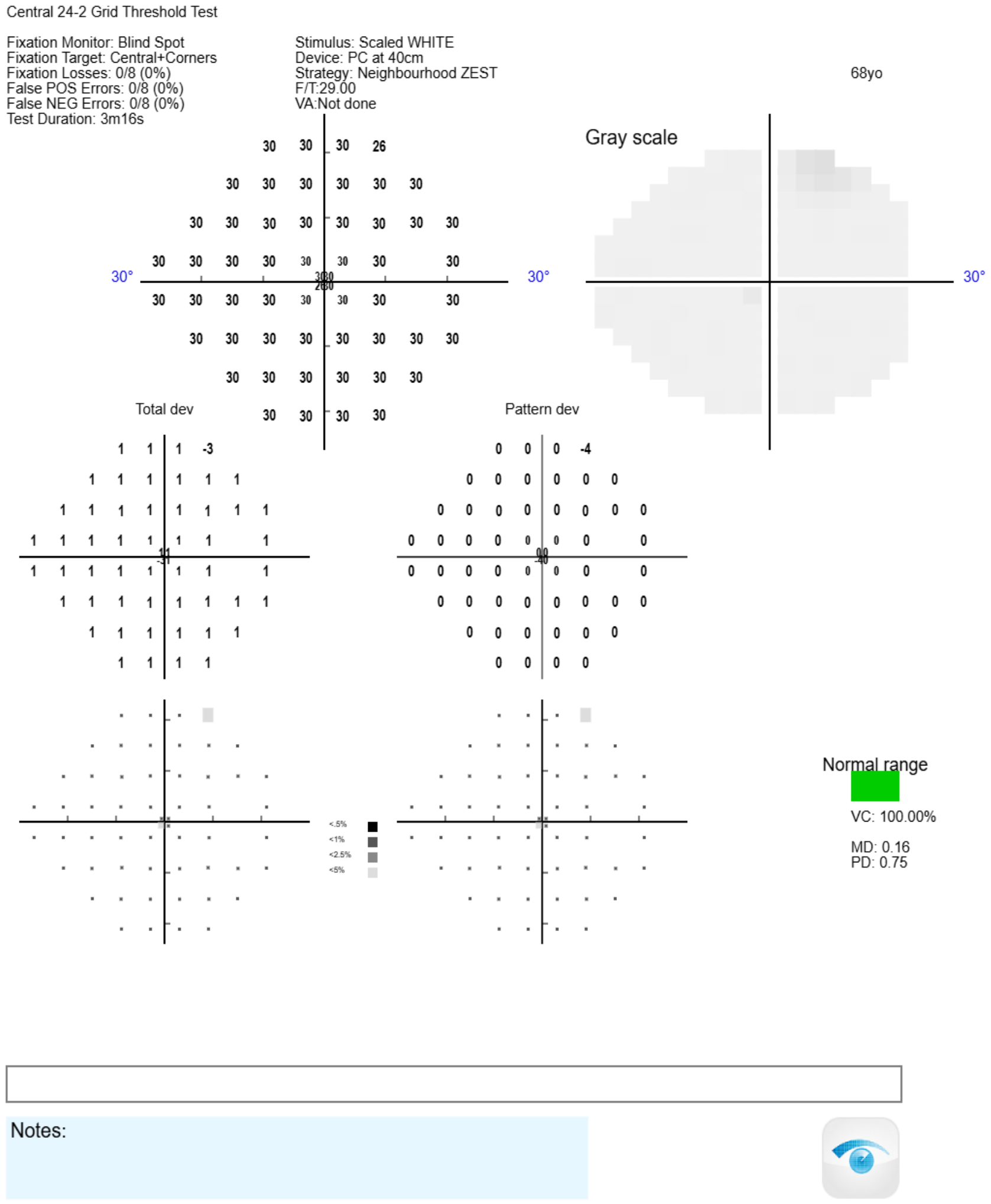

Supplement: Supplementary file 1 [file Image1.pdf]
